# Supplementary material for: Synthesis and Validation of TRIFAPYs as a Family of Transfection Agents for Therapeutic Oligonucleotides
Source: Biomolecules. 2024 Mar 25;14(4):390. doi: 10.3390/biom14040390 (PMC11048556; doi:10.3390/biom14040390)
Supplement: Supplementary file 1 [file biomolecules-14-00390-s001.zip › biomolecules-2895273-supplementary.pdf]

Supplementary data

Supporting Information Available

- Espectroscopic data for alkoxypyridines **1a-1i**
- ^{1}H and ^{13}C NMR spectra of compounds **1a-1i, 2a-2i and 3.**

4-Butoxypyridine (1a): ^{1}H NMR (400 MHz, $CDCl_3$) δ 8.40 (d, *J* = 5.0 Hz, 2H), 6.79 (d, *J* = 5.0 Hz, 2H), 4.01 (t, *J* = 6.5 Hz, 2H), 1.82-1.75 (m, 2H), 1.49 (sex, *J* = 7.4 Hz, 2H), 0.98 (t, *J* = 7.4 Hz, 3H). ^{13}C NMR (100.6 MHz, $CDCl_3$) δ 165.2 (C), 151.1 (CH), 110.4 (CH), 67.7 (CH_2), 31.0 (CH_2), 19.2 (CH_2), 13.9 (CH_3).

4-Hexyloxypyridine (1b): ^{1}H NMR (400 MHz, $CDCl_3$) δ 8.41 (d, *J* = 5.2 Hz, 2H), 6.79 (d, *J* = 5.2 Hz, 2H), 4.00 (t, *J* = 6.5 Hz, 2H), 1.83-1.76 (m, 2H), 1.49-1.42 (m, 2H), 1.36-1.32 (m, 4H), 0.91 (t, *J* = 7.0 Hz, 3H). ^{13}C NMR (100.6 MHz, $CDCl_3$) δ 165.1(C), 150.9 (CH), 110.3 (CH), 67.9 (CH_2), 31.5 (CH_2), 28.8 (CH_2), 25.6 (CH_2), 22.5 (CH_2), 14.0 (CH_3).

4-Dodecyloxypyridine (1e): ^{1}H NMR (400 MHz, $CDCl_3$) δ 8.41 (d, *J* = 5.0 Hz, 2H), 6.80 (d, *J* = 5.0 Hz, 2H), 4.00 (t, *J* = 6.6 Hz, 2H), 1.83-1.76 (m, 2H), 1.49-1.41 (m, 2H), 1.37-1.24 (m, 16H), 0.88 (t, *J* = 6.7 Hz, 3H). ^{13}C NMR (100.6 MHz, $CDCl_3$) δ 164.9 (C), 158.8 (CH), 110.0 (CH), 67.6 (CH_2), 31.8 (CH_2), 29.6 (CH_2), 29.5 (CH_2), 29.5 (CH_2), 29.4(CH_2), 29.2 (CH_2), 29.1 (CH_2), 28.7 (CH_2), 25.8 (CH_2), 22.5 (CH_2), 14.0 (CH_3).

4-Tetradecyloxypyridine (1f): ^{1}H NMR (400 MHz, $CDCl_3$) δ 8.41 (d, *J* = 5.2 Hz, 2H), 6.79 (d, *J* = 5.2 Hz, 2H), 3.99 (t, *J* = 6.6 Hz, 2H), 1.83-1.76 (m, 2H), 1.48-1.41 (m, 2H), 1.36-1.24 (m, 20H), 0.87 (t, *J* = 7 Hz, 3H). ^{13}C NMR (100.6 MHz, $CDCl_3$) δ 165.1 (C), 151.0 (CH), 110.3 (CH), 67.9 (CH_2), 31.9 (CH_2), 29.7 (CH_2), 29.6 (CH_2), 29.6 (CH_2), 29.5 (CH_2), 29.5 (CH_2), 29.3 (CH_2), 29.3 (CH_2), 28.9 (CH_2), 25.9 (CH_2), 22.7 (CH_2), 14.1 (CH_3).

4-Octyloxypyridine (1c): ^{1}H NMR (400 MHz, $CDCl_3$) δ 8.41 (d, *J* = 6.4 Hz, 2H), 6.78 (d, *J* = 6.4 Hz, 2H), 4.00 (t, *J* = 6.5 Hz, 2H), 1.83-1.76 (m, 2H), 1.49-1.41 (m, 2H), 1.34-1.29 (m, 8H), 0.90-0.87 (m, 3H). ^{13}C NMR (100.6 MHz, $CDCl_3$) δ 164.9 (C), 150.8 (CH), 110.1 (CH), 67.7 (CH_2), 31.6 (CH_2), 29.1 (CH_2), 29.0 (CH_2), 28.7(CH_2), 25.8 (CH_2), 22.5 (CH_2), 13.9 (CH_3).

4-Decyloxypyridine (1d): ^{1}H NMR (400 MHz, $CDCl_3$) δ 8.39 (d, *J* = 4.9 Hz, 2H), 6.76 (d, *J* = 4.9 Hz, 2H), 3.96 (t, *J* = 6.6 Hz, 2H), 1.81-1.74 (m, 2H), 1.47-1.41 (m, 2H), 1.36-1.25 (m, 12H), 0.90-0.86 (m, 3H). ^{13}C NMR (100.6 MHz, $CDCl_3$) δ 165.0 (C), 151.0 (CH), 110.2 (CH), 67.8 (CH_2), 31.8 (CH_2), 29.5 (CH_2), 29.3 (CH_2), 29.2 (CH_2), 28.8 (CH_2), 25.9 (CH_2), 22.6 (CH_2), 14.0 (CH_3).

4-Hexadecyloxypyridine (1g): ^{1}H NMR (400 MHz, $CDCl_3$) δ 8.41 (d, *J* = 5.0 Hz, 2H), 6.78 (d, *J* = 5.0 Hz, 2H), 3.98 (t, *J* = 6.5 Hz, 2H), 1.85-1.73 (m, 2H), 1.50-1.33 (m, 2H), 1.36-1.24 (m, 24H), 0.88 (t, *J* = 6.6 Hz, 3H). ^{13}C NMR (100.6 MHz, $CDCl_3$) δ 165.1 (C), 150.9 (CH), 110.3 (CH), 67.9 (CH_2), 31.9 (CH_2), 29.7 (CH_2), 29.7 (CH_2), 29.7 (CH_2), 29.6 (CH_2), 29.5 (CH_2), 29.4 (CH_2), 29.3 (CH_2), 28.9 (CH_2), 25.9 (CH_2), 22.7 (CH_2), 14.1 (CH_3).

4-Octadecyloxypyridine (1h): ^{1}H NMR (400 MHz, $CDCl_3$) δ 8.40 (d, *J* = 6.5 Hz, 2H), 6.77 (d, *J* = 6.5 Hz, 2H), 3.98 (t, *J* = 6.5 Hz, 2H), 1.82-1.75 (m, 2H), 1.48-1.41 (m, 2H), 1.34-1.23 (m, 28H), 0.88 (t, *J* = 6.8 Hz, 3H).

4-Eicosanoyloxypyridine (1i): ^{1}H NMR (400 MHz, $CDCl_3$) δ 8.34 (d, *J* = 6.0 Hz, 2H), 6.73 (d, *J* = 6.0 Hz, 2H), 3.96 (t, *J* = 6.6 Hz, 2H), 1.76-1.69 (m, 2H), 1.41-1.34 (m, 2H), 1.29-1.15 (m, 32H), 0.80 (t, *J* = 7 Hz, 3H). ^{13}C NMR (100.6 MHz, $CDCl_3$) δ 165.3 (C), 150.7 (CH), 110.3 (CH), 68.0 (CH_2), 31.9 (CH_2), 29.7 (CH_2), 29.6 (CH_2), 29.6 (CH_2), 29.5 (CH_2), 29.5 (CH_2), 29.3 (CH_2), 29.3 (CH_2), 28.8 (CH_2), 25.9 (CH_2), 22.7 (CH_2), 14.1 (CH_3).

A.

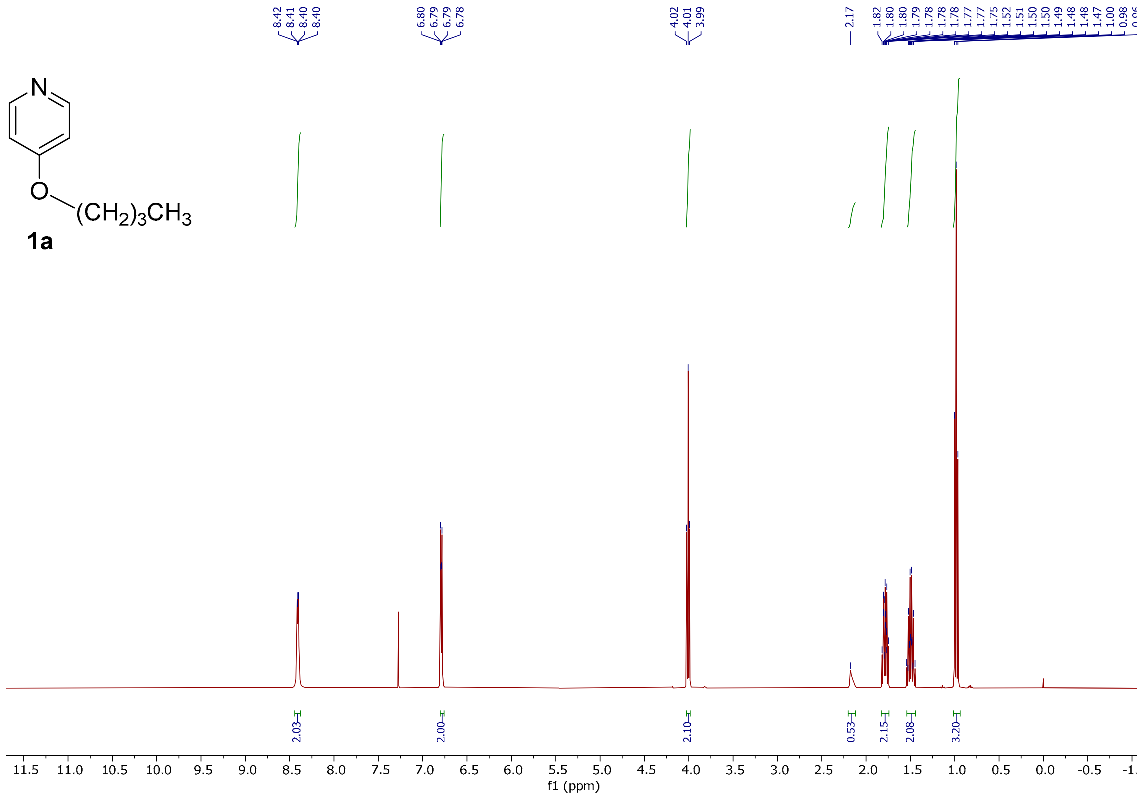

B.

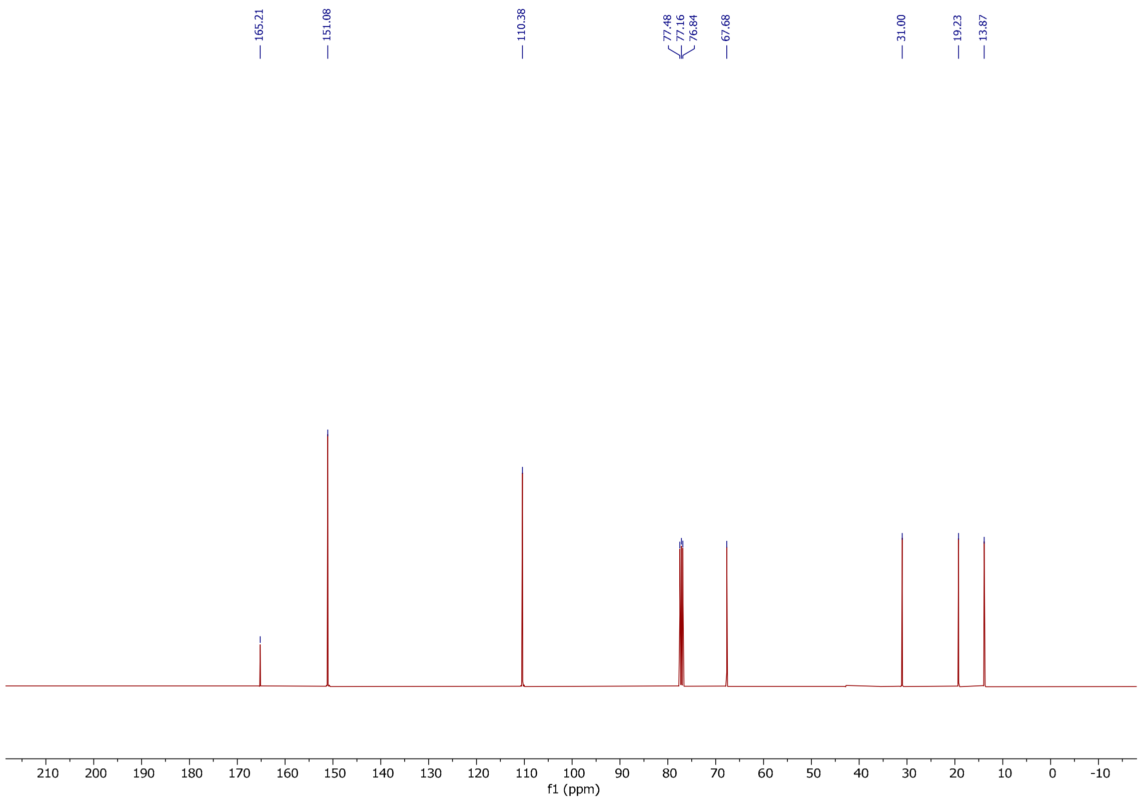

Figure S1. (A) ^{1}H-NMR (400 MHz, $CDCl_3$) and (B) ^{13}C NMR (100.6 MHz, $CDCl_3$) spectra of 4-butoxypyridine.

A.

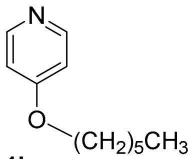

1b

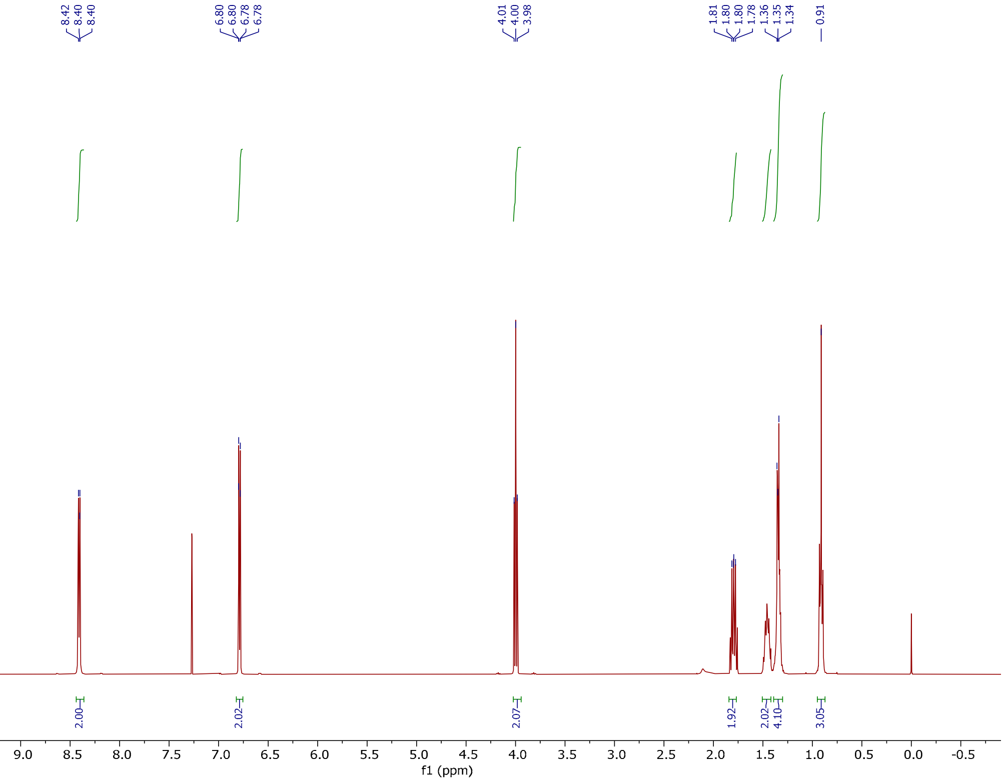

B.

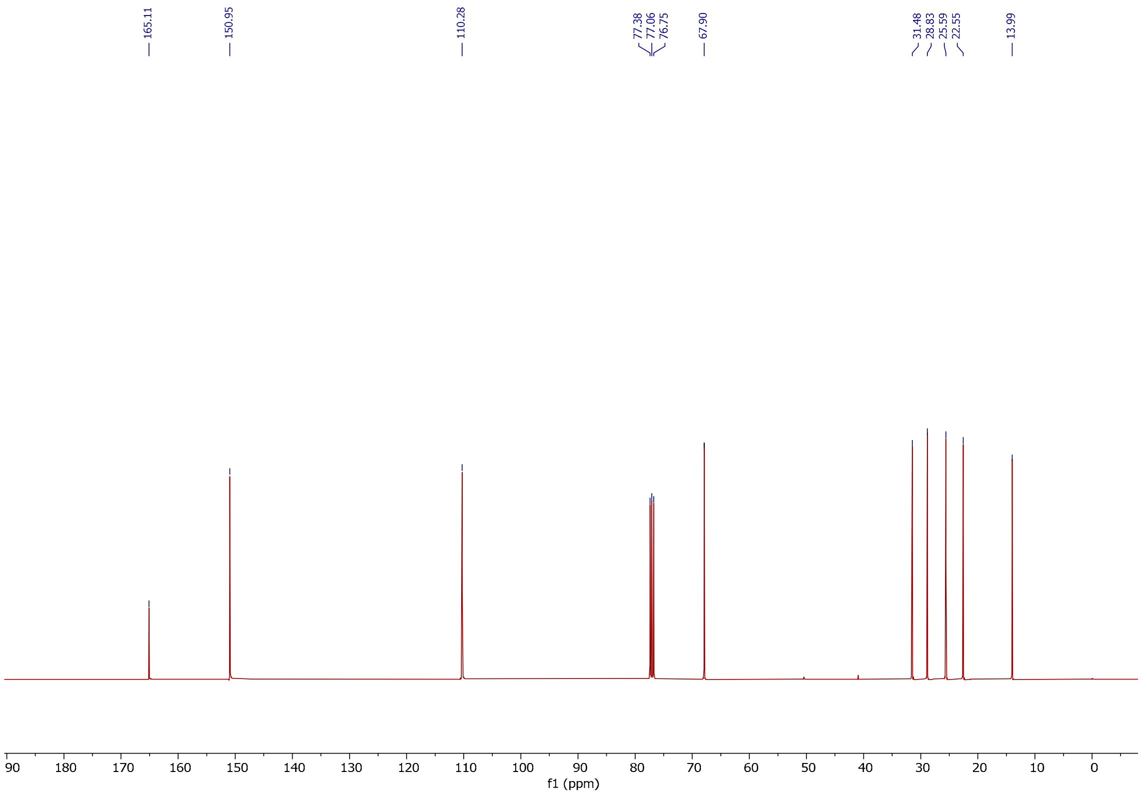

Figure S2. (A) ^{1}H-NMR (400 MHz, $CDCl_3$) and (B) ^{13}C NMR (100.6 MHz, $CDCl_3$) spectra of 4-Hexyloxypyridine.

A.

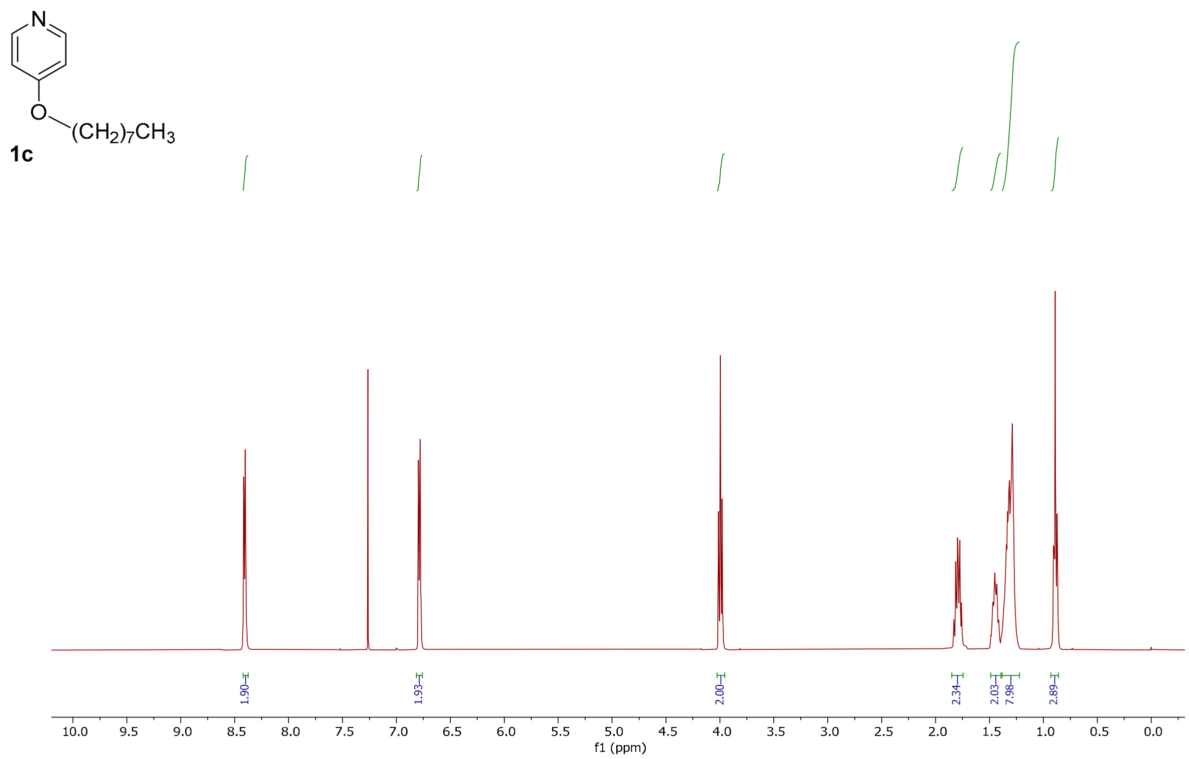

B.

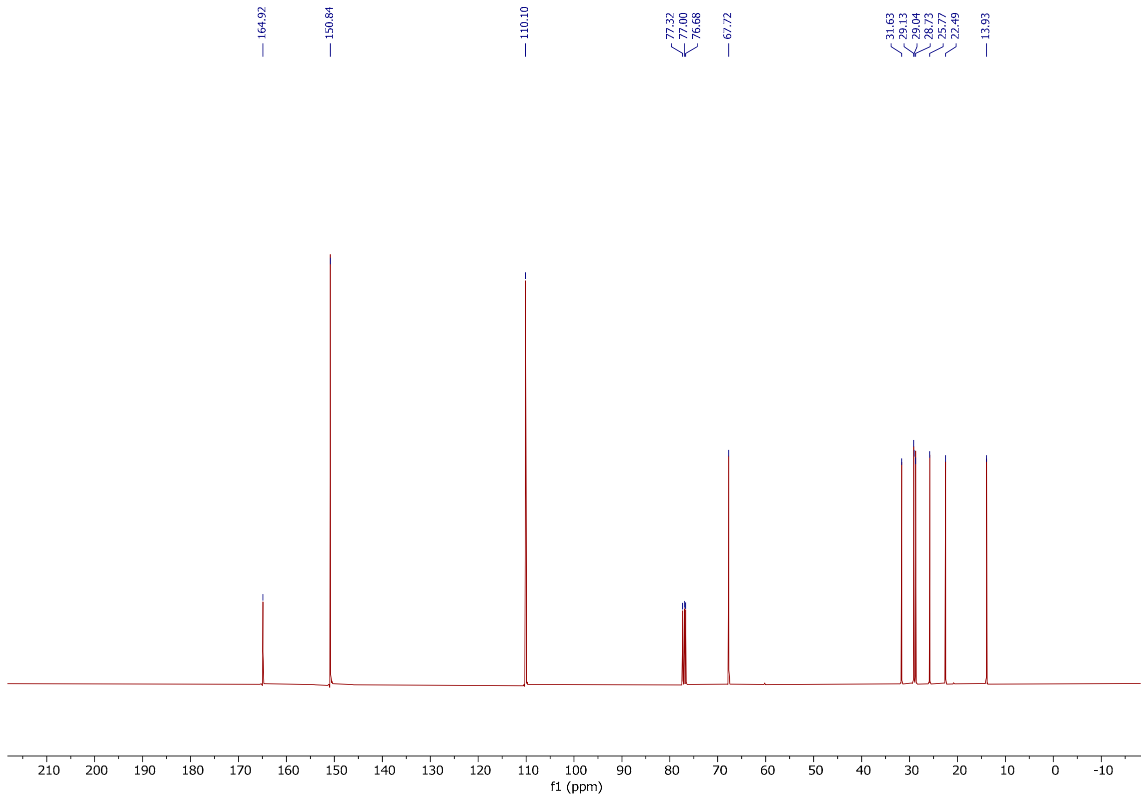

Figure S3. (A) ^{1}H-NMR (400 MHz, $CDCl_3$) and (B) ^{13}C NMR (100.6 MHz, $CDCl_3$) spectra of 4-Octyloxypyridine.

A.

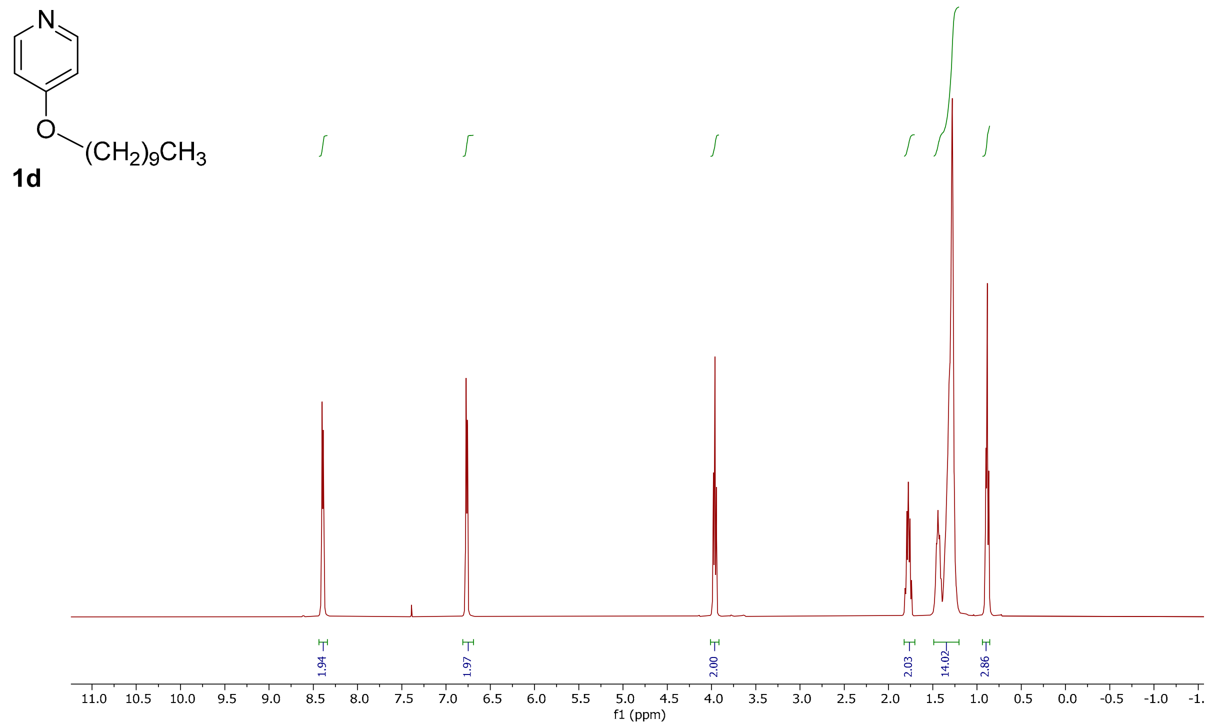

B.

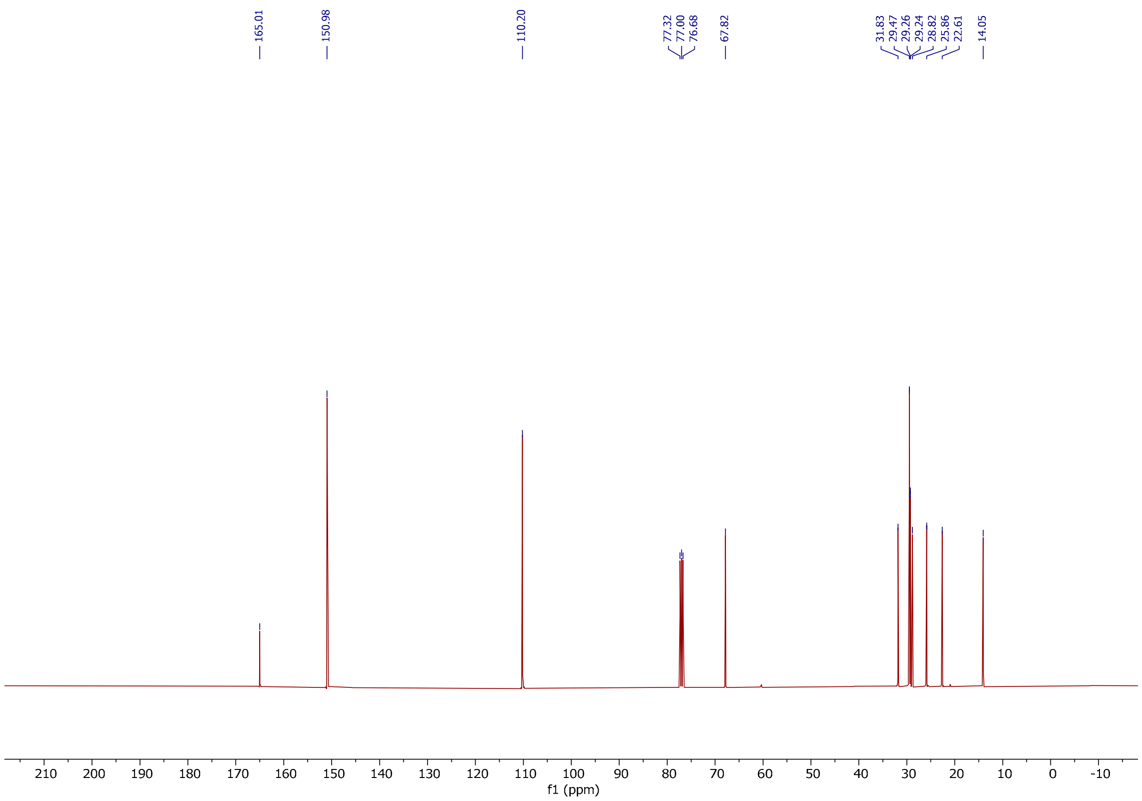

Figure S4. (A) ^{1}H-NMR (400 MHz, $CDCl_3$) and (B) ^{13}C NMR (100.6 MHz, $CDCl_3$) spectra of 4-Decyloxypyridine.

A.

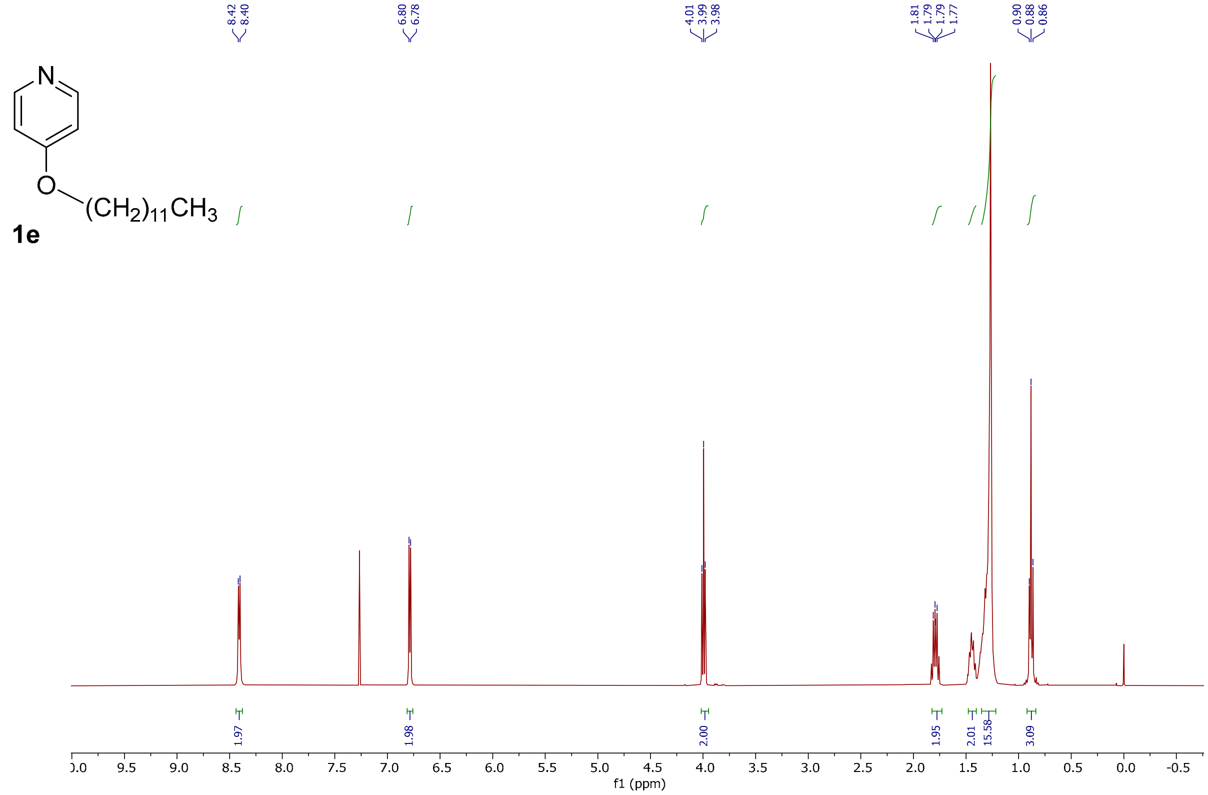

B.

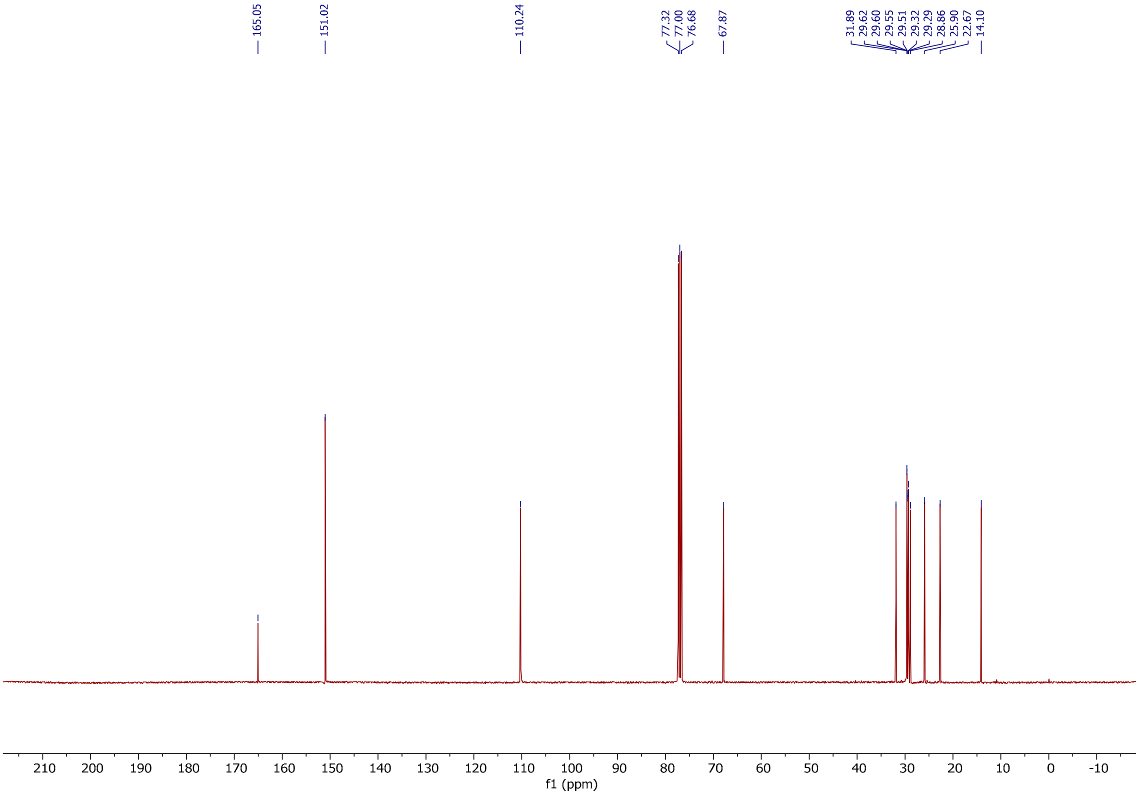

Figure S5. (A) ^{1}H-NMR (400 MHz, $CDCl_3$) and (B) ^{13}C NMR (100.6 MHz, $CDCl_3$) spectra of 4-Dodecyloxypyridine.

A.

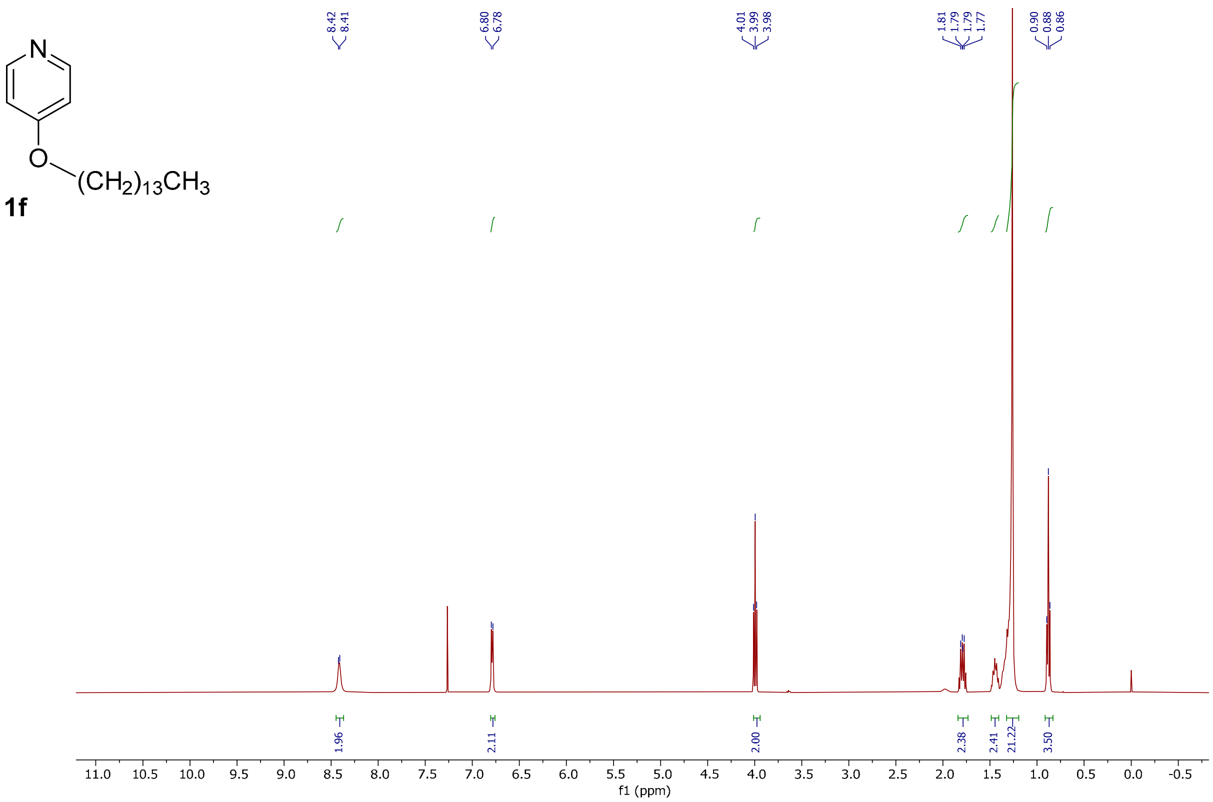

B.

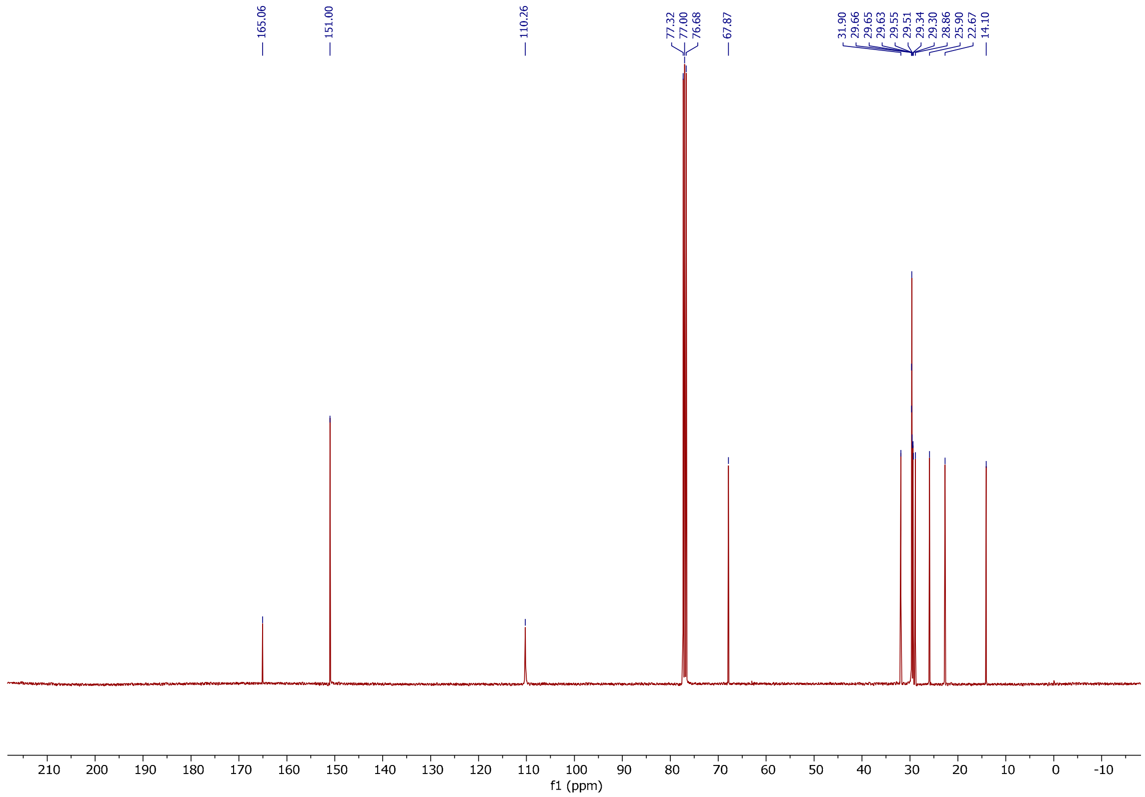

Figure S6. (A) ^{1}H-NMR (400 MHz, $CDCl_3$) and (B) ^{13}C NMR (100.6 MHz, $CDCl_3$) spectra of 4-Tetradecyloxypyridine.

A.

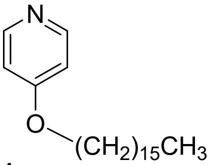

1g

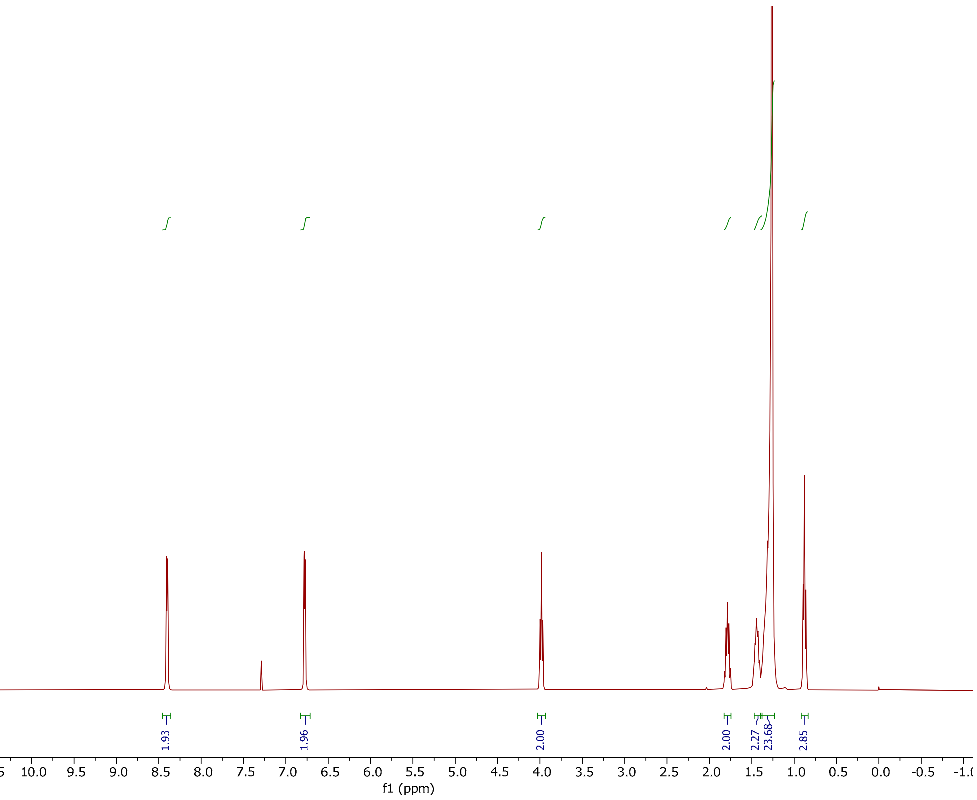

B.

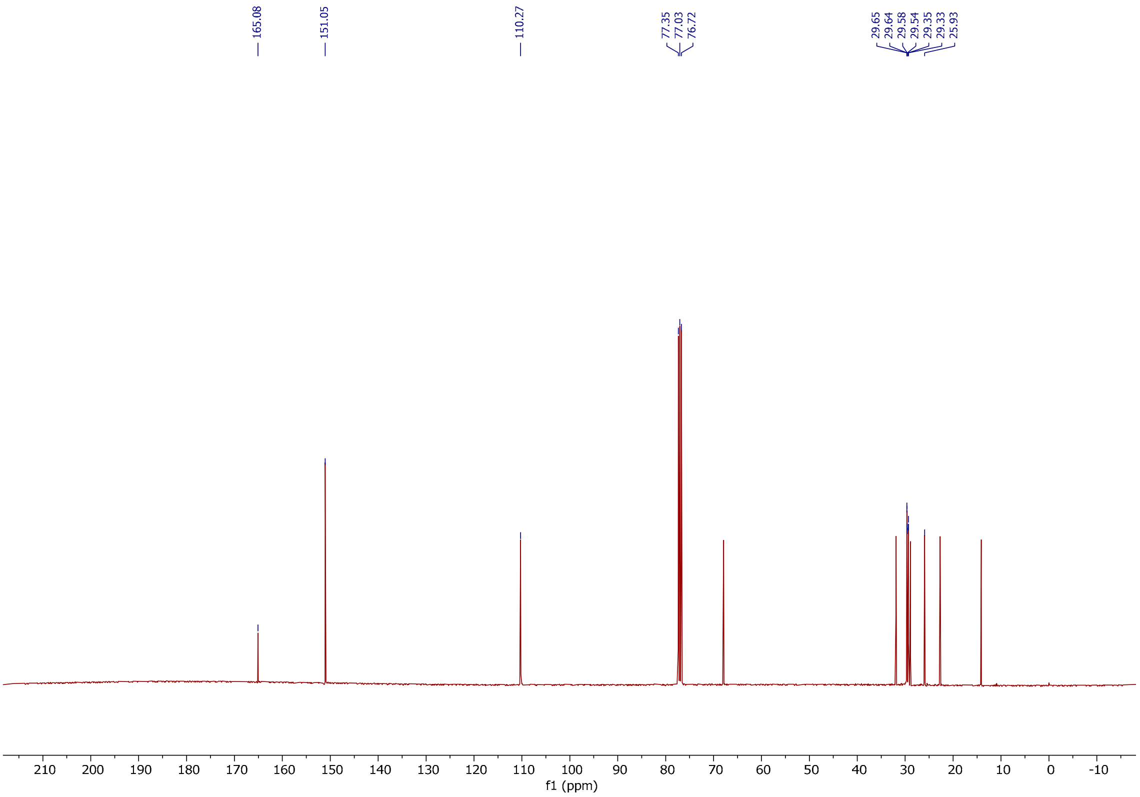

Figure S7. (A) ^{1}H-NMR (400 MHz, $CDCl_3$) and (B) ^{13}C NMR (100.6 MHz, $CDCl_3$) spectra of 4-Hexadecyloxypyridine.

A.

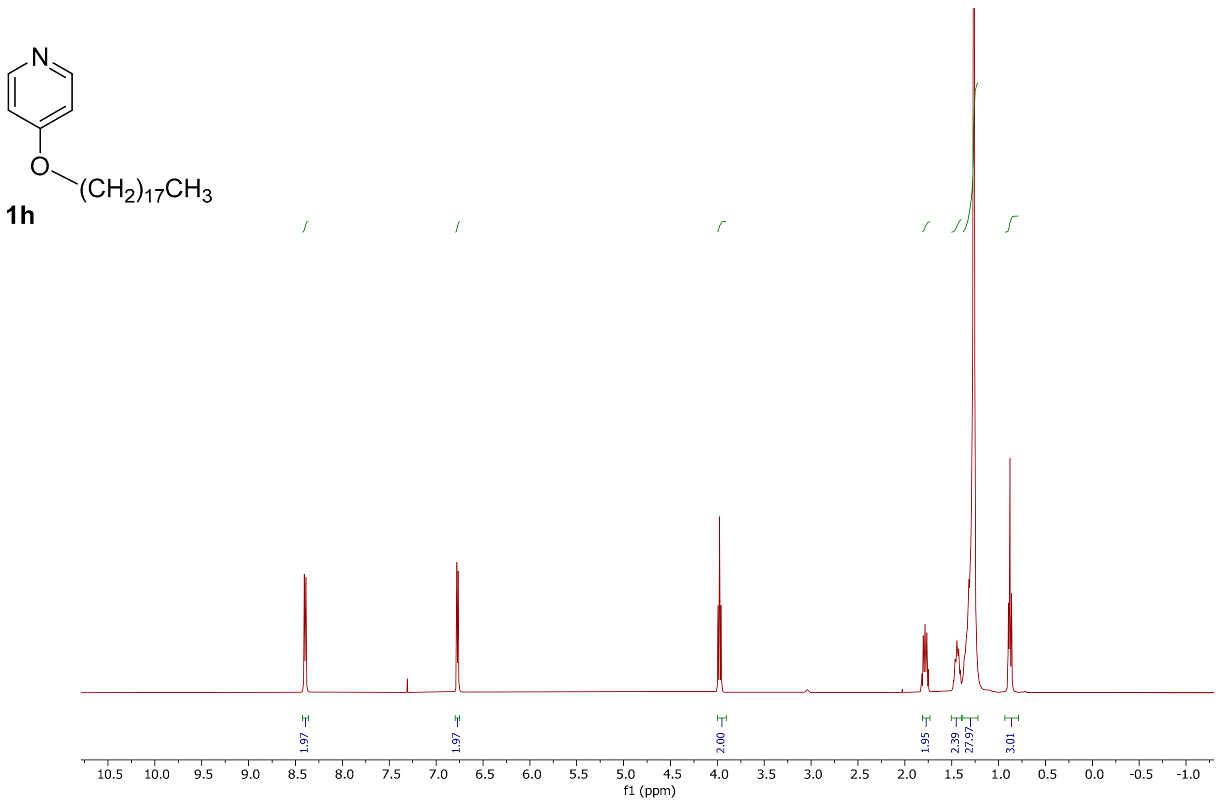

Figure S8. (A) ^{1}H-NMR (400 MHz, $CDCl_3$) spectra of 4-Octadecyloxypyridine.

A.

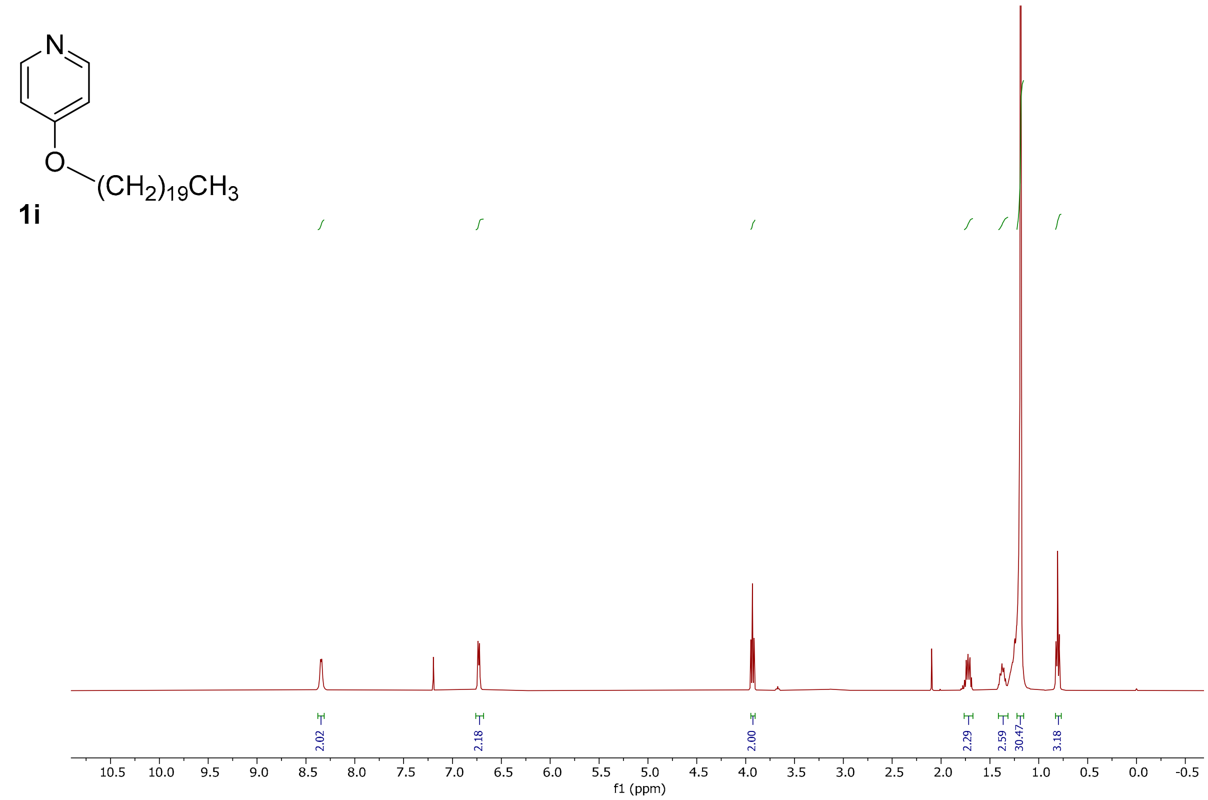

B.

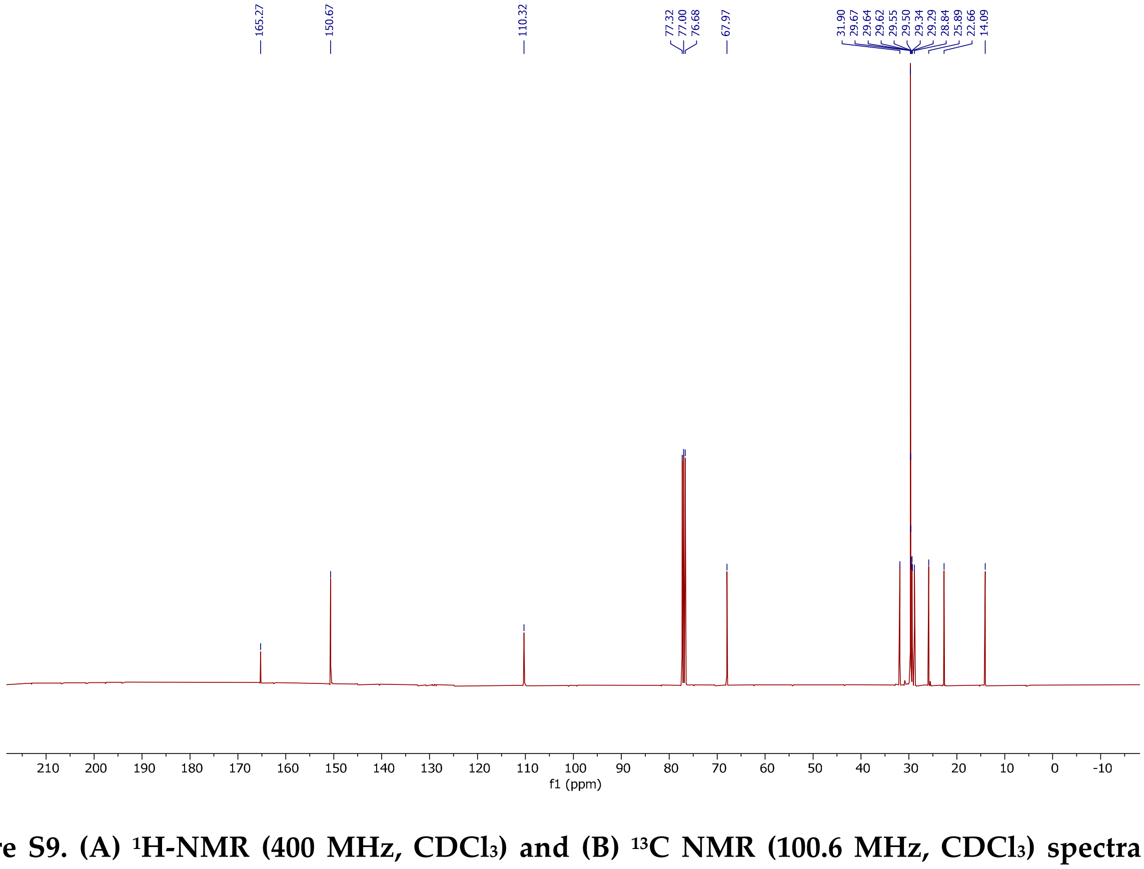

Figure S9. **(A)** ^{1}**H-NMR (400 MHz,** $CDCl_3$**) and (B)** ^{13}**C NMR (100.6 MHz,** $CDCl_3$**) spectra of 4-Eicosanoyloxypyridine.**

A.

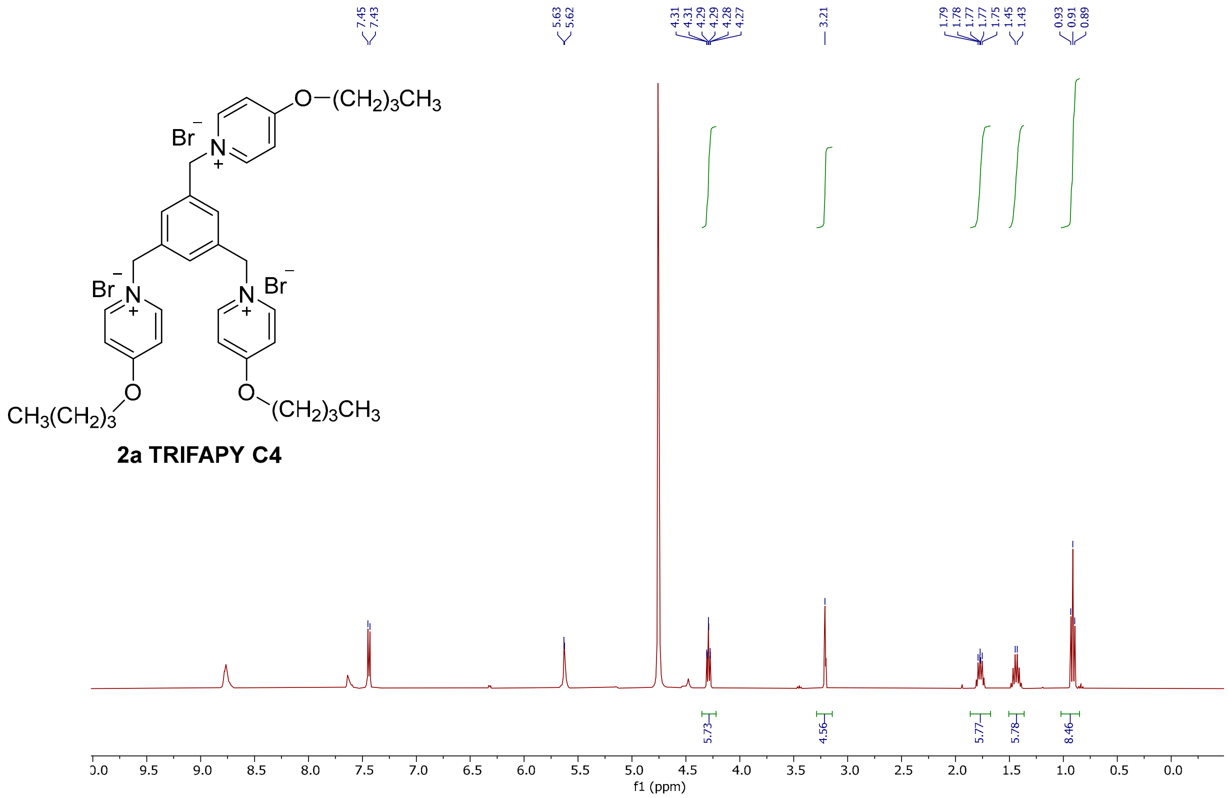

B.

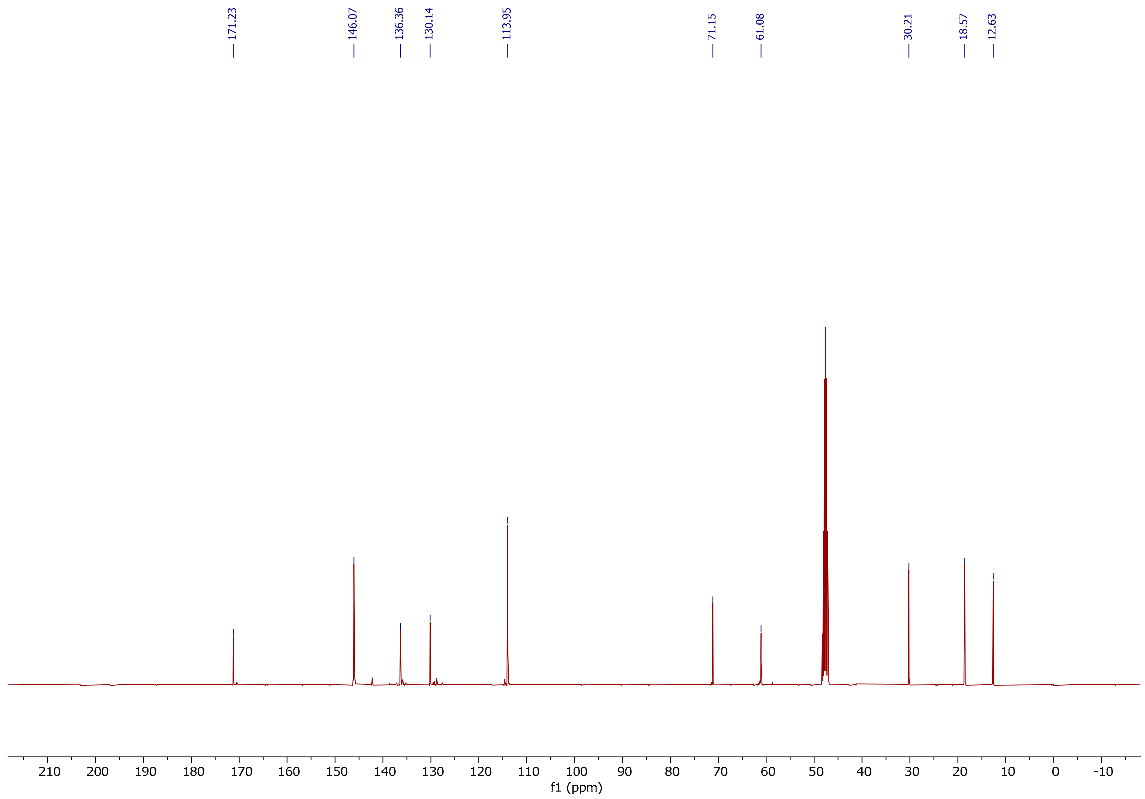

Figure S10. (A) ^{1}H-NMR (400 MHz, CD_3OD) and (B) ^{13}C NMR (100.6 MHz, CD_3OD) spectra of 1,3,5-Tris(4-butoxypyridiniomethyl)benzene tribromide.

A.

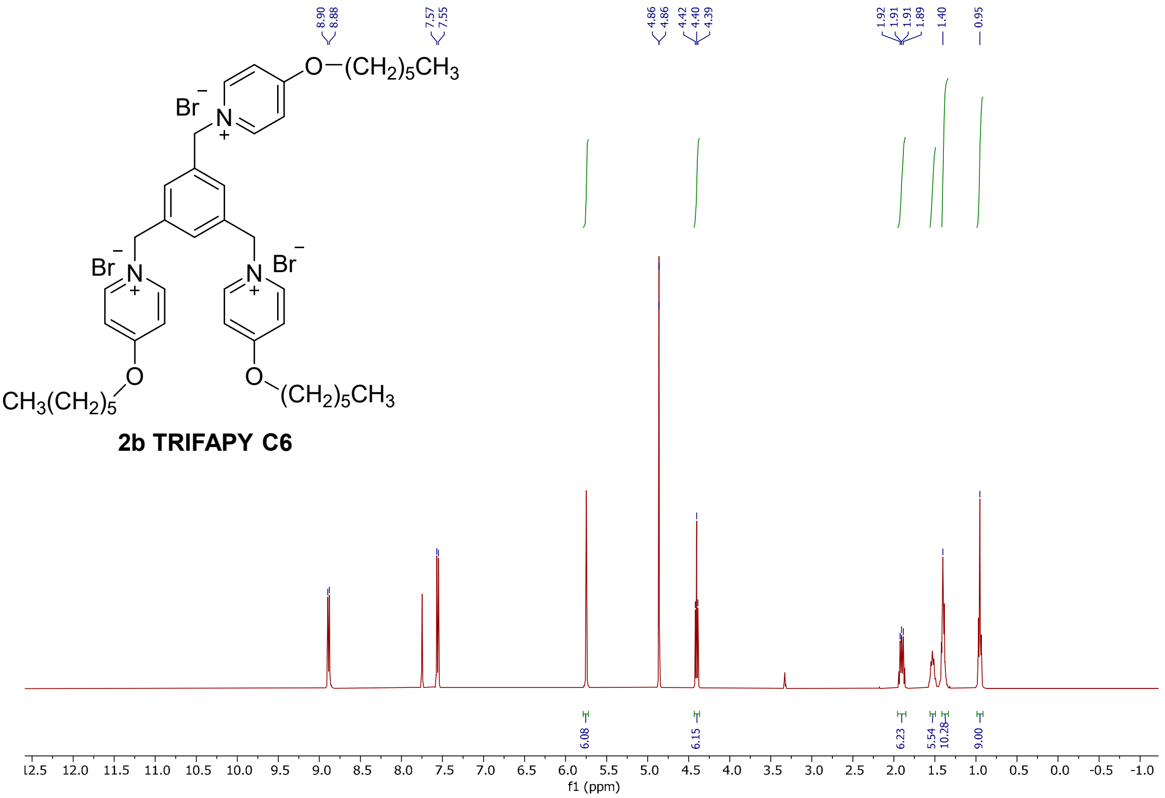

B.

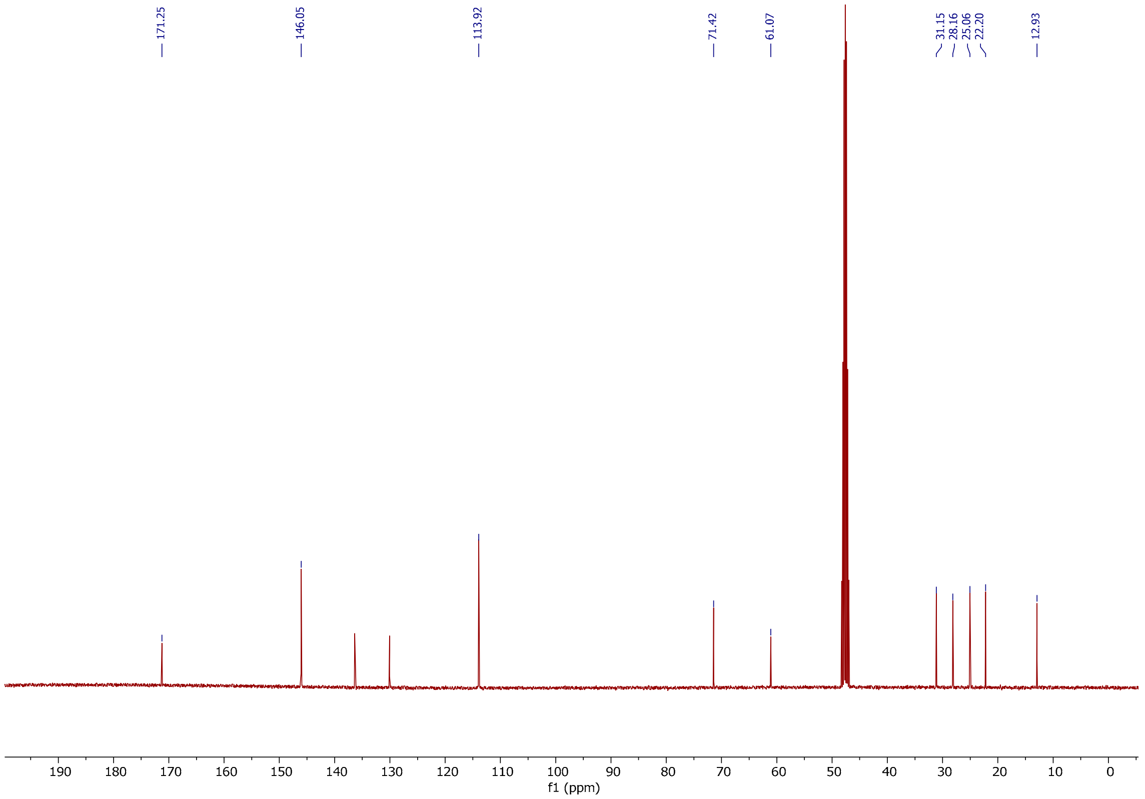

Figure S11. (A) ^{1}H-NMR (400 MHz, CD_3OD) and (B) ^{13}C NMR (100.6 MHz, CD_3OD) spectra of 1,3,5-Tris(4-hexyloxypyridiniomethyl)benzene tribromide.

A.

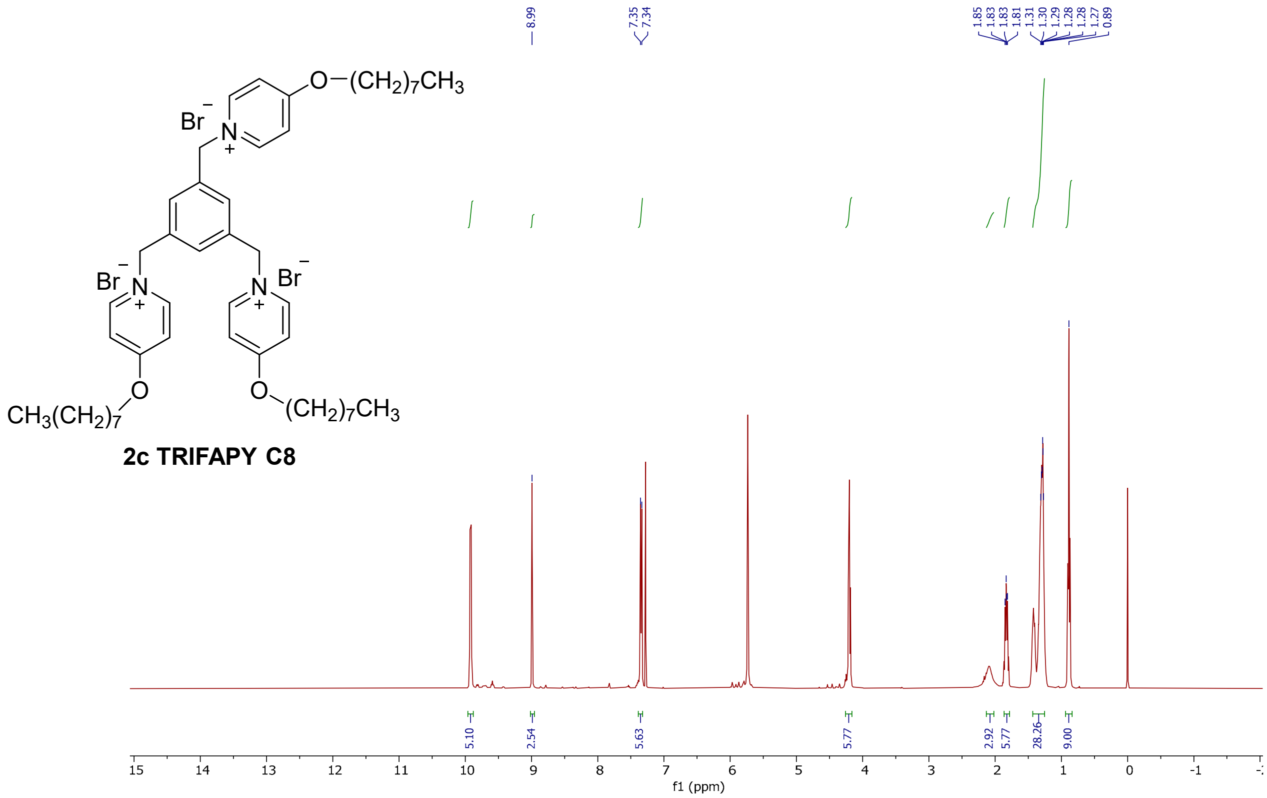

B.

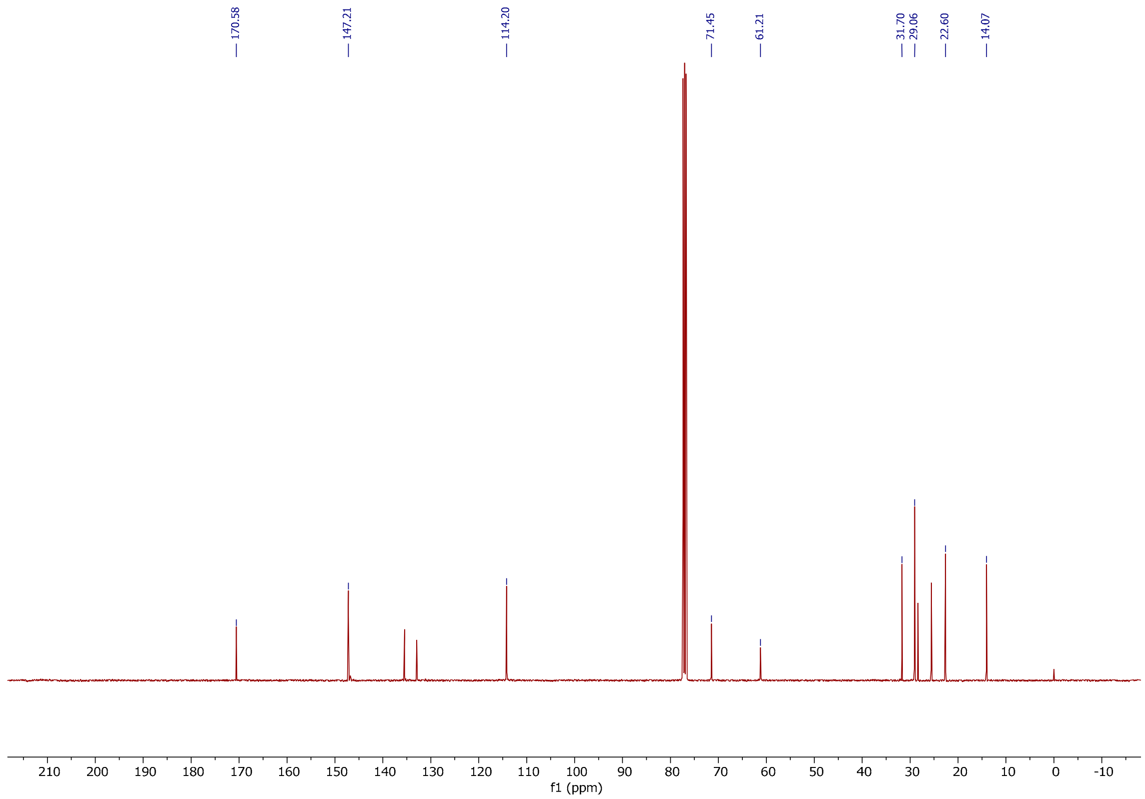

Figure S12. (A) ^{1}H-NMR (400 MHz, $CDCl_3$) and (B) ^{13}C NMR (100.6 MHz, $CDCl_3$) spectra of 1,3,5-Tris(4-octyloxypyridiniomethyl)benzene tribromide.

A.

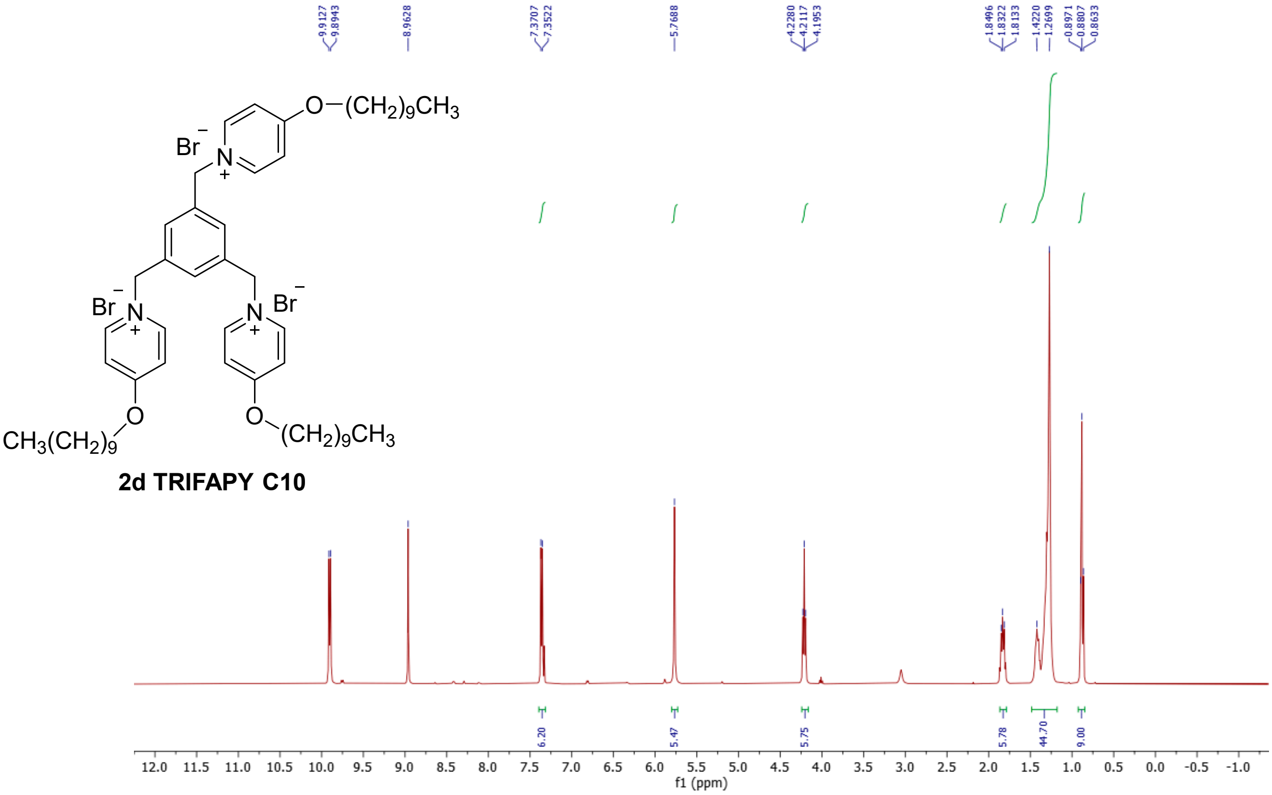

B.

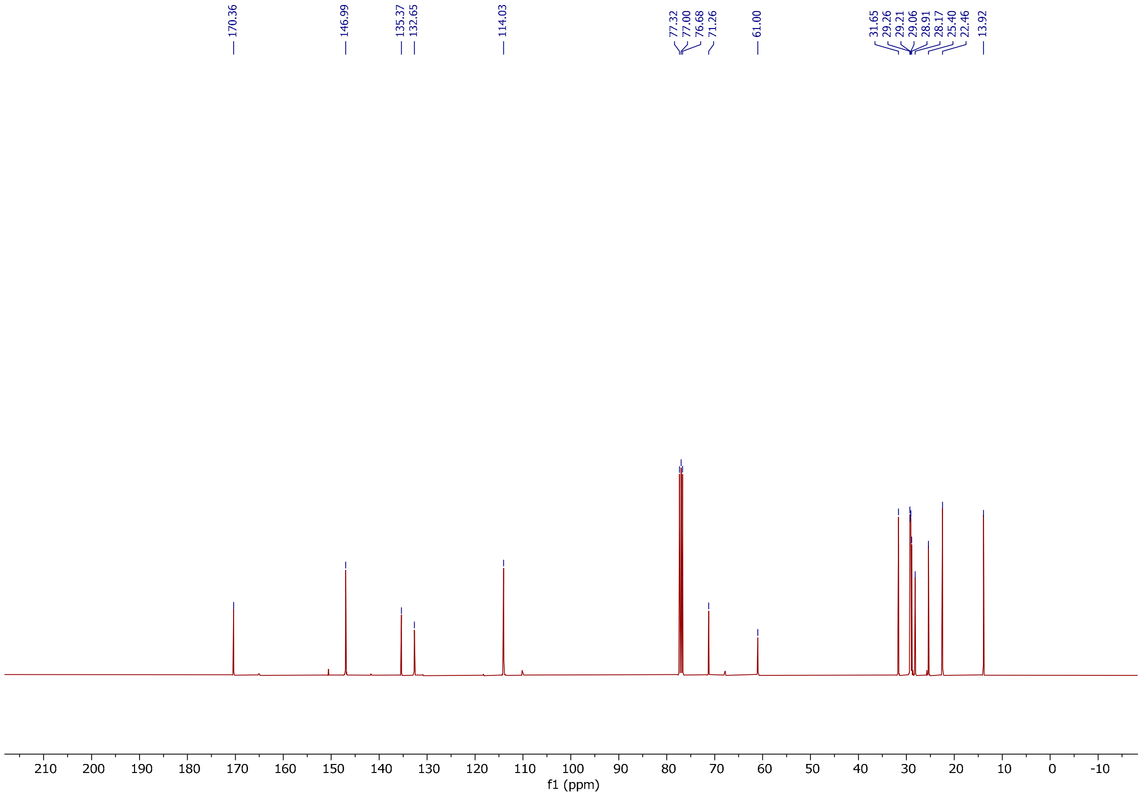

Figure S13. (A) ¹H-NMR (400 MHz, CDCl₃) and (B) ¹³C NMR (100.6 MHz, CDCl₃) spectra of 1,3,5-Tris(4-decyloxypyridiniomethyl)benzene tribromide.

A.

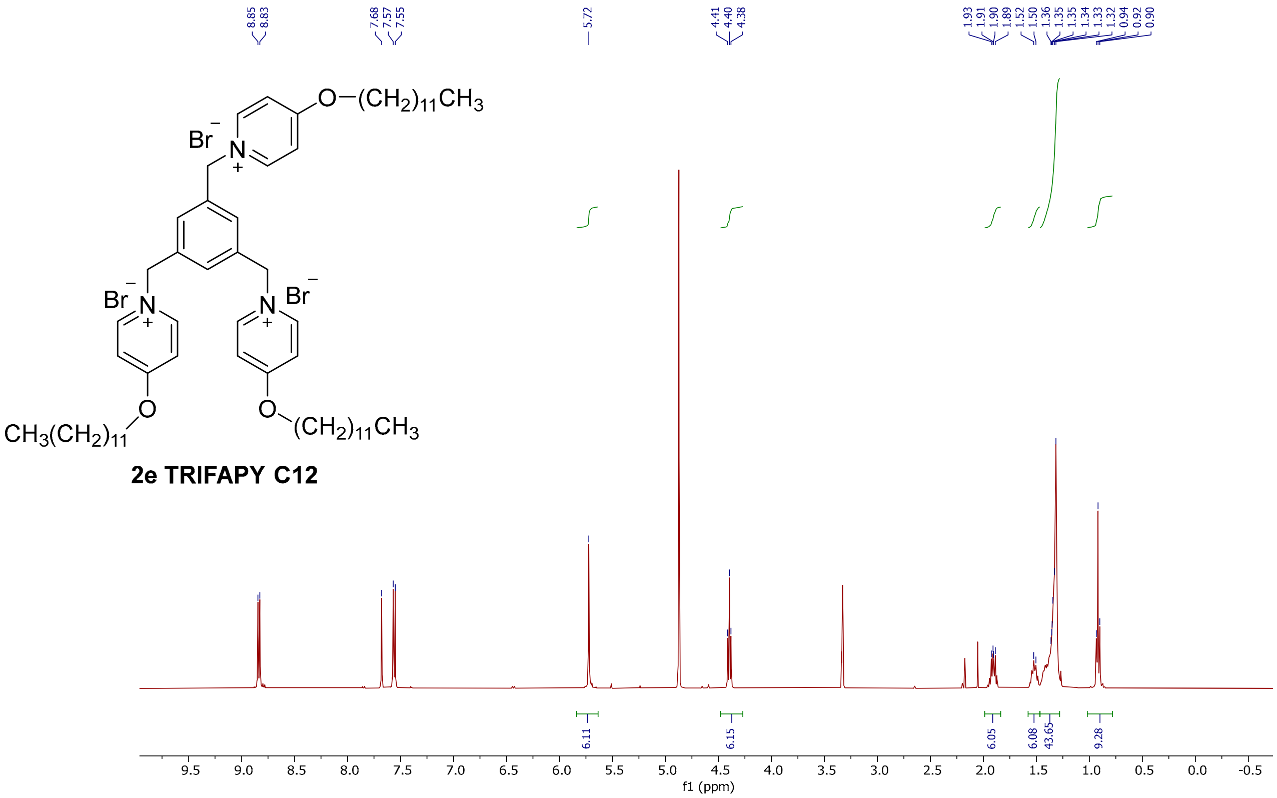

B.

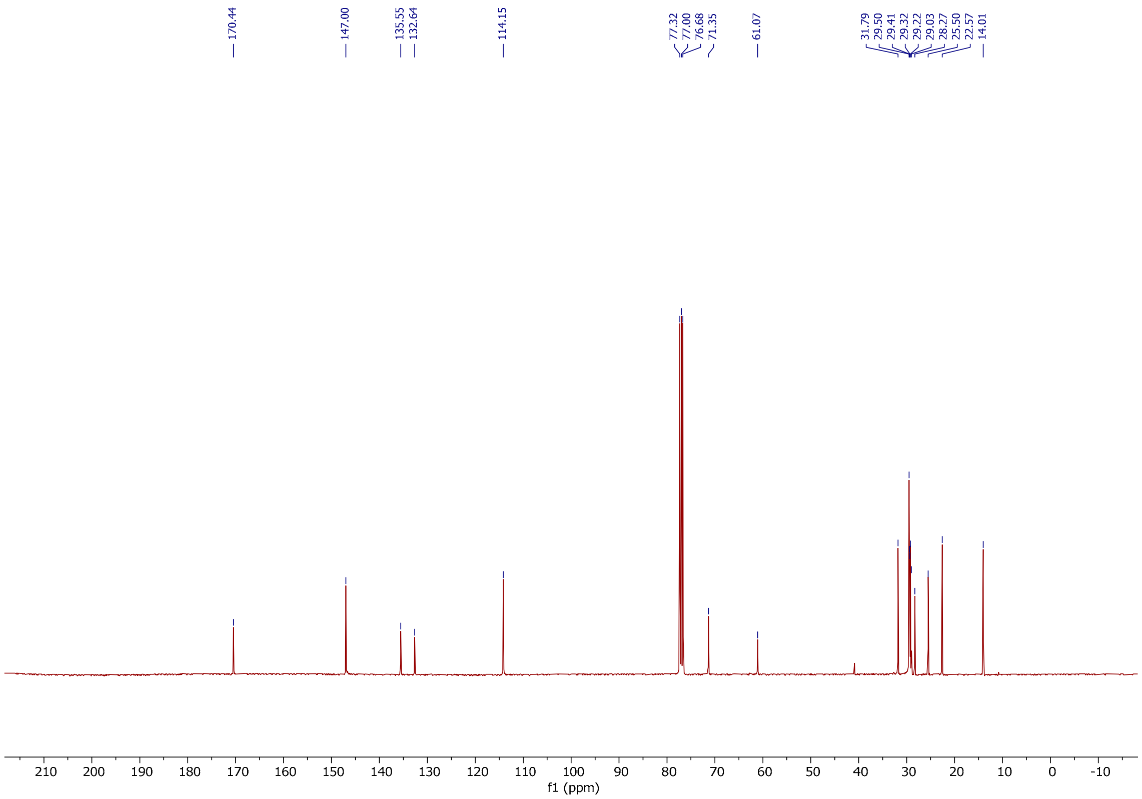

Figure S14. (A) ^{1}H-NMR (400 MHz, CD_3OD) and (B) ^{13}C NMR (100.6 MHz, CD_3OD) spectra of 1,3,5-Tris(4-dodecyloxypyridiniomethyl)benzene tribromide.

A.

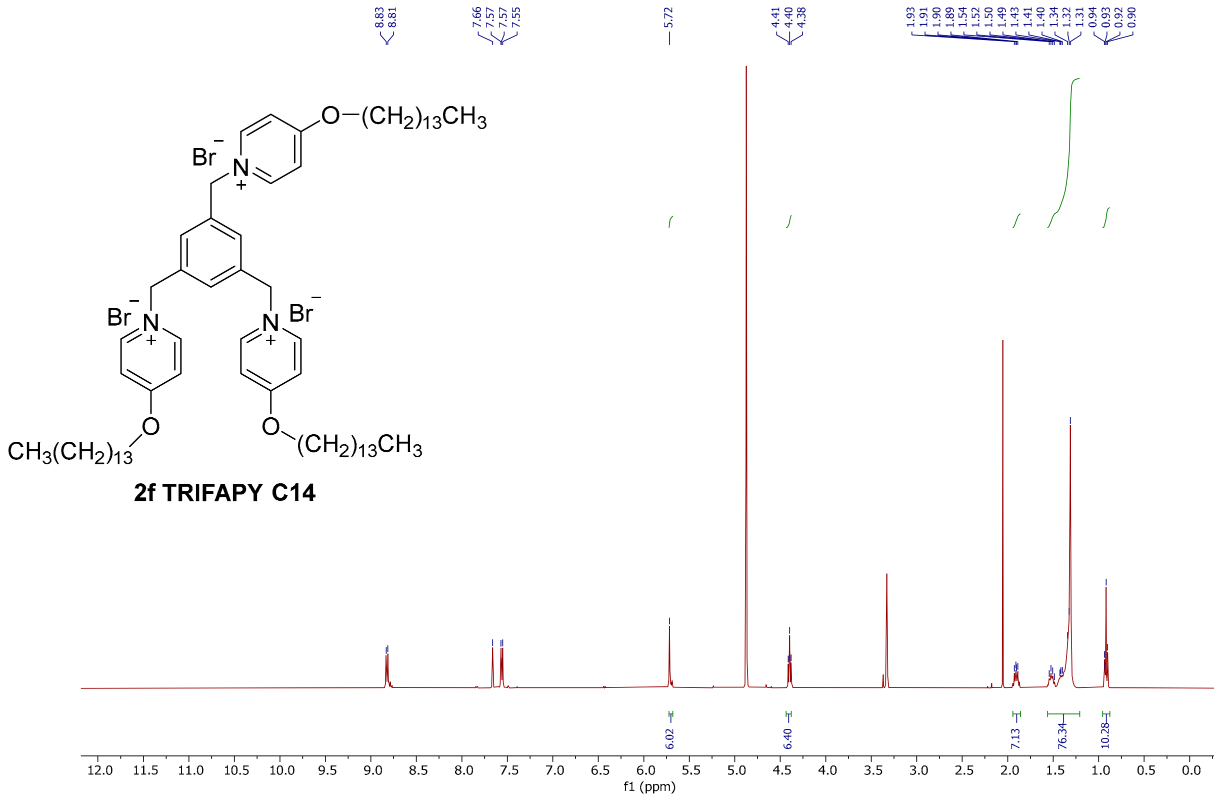

B.

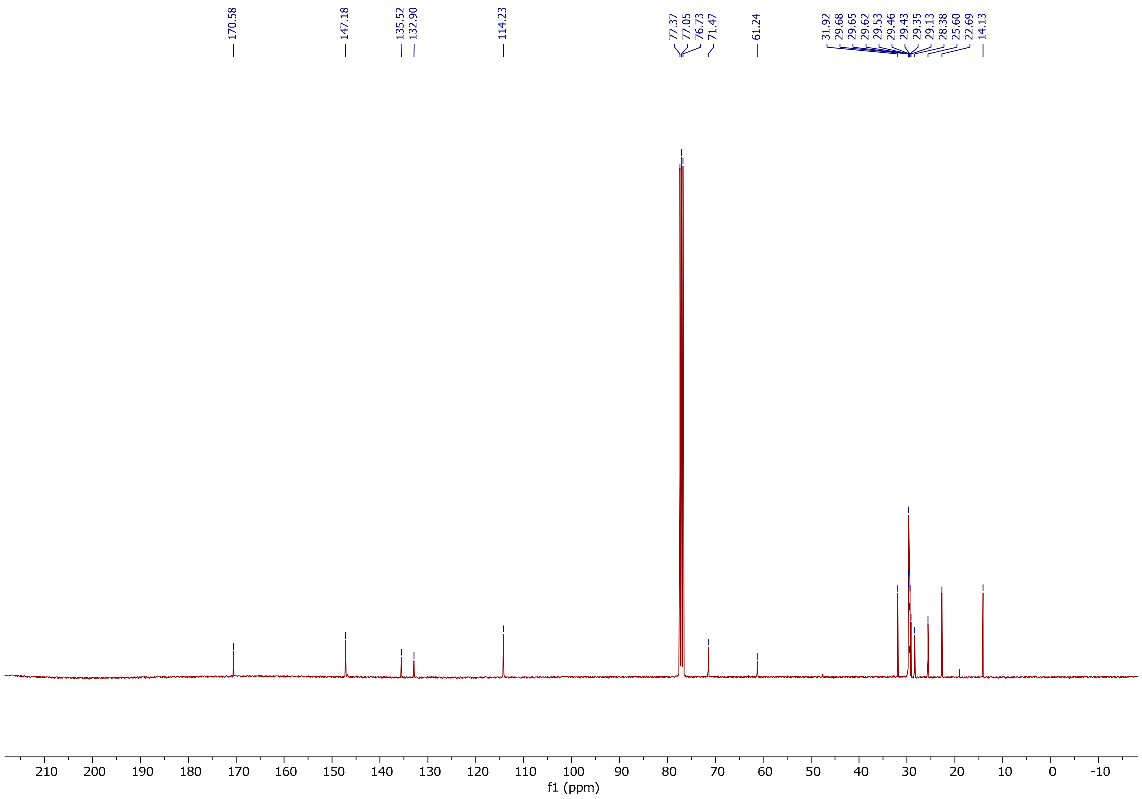

Figure S15. (A) ^{1}H-NMR (400 MHz, CD_3OD) and (B) ^{13}C NMR (100.6 MHz, $CDCl_3$) spectra of 1,3,5-Tris(4-tetradecyloxypyridiniomethyl)benzene tribromide.

A.

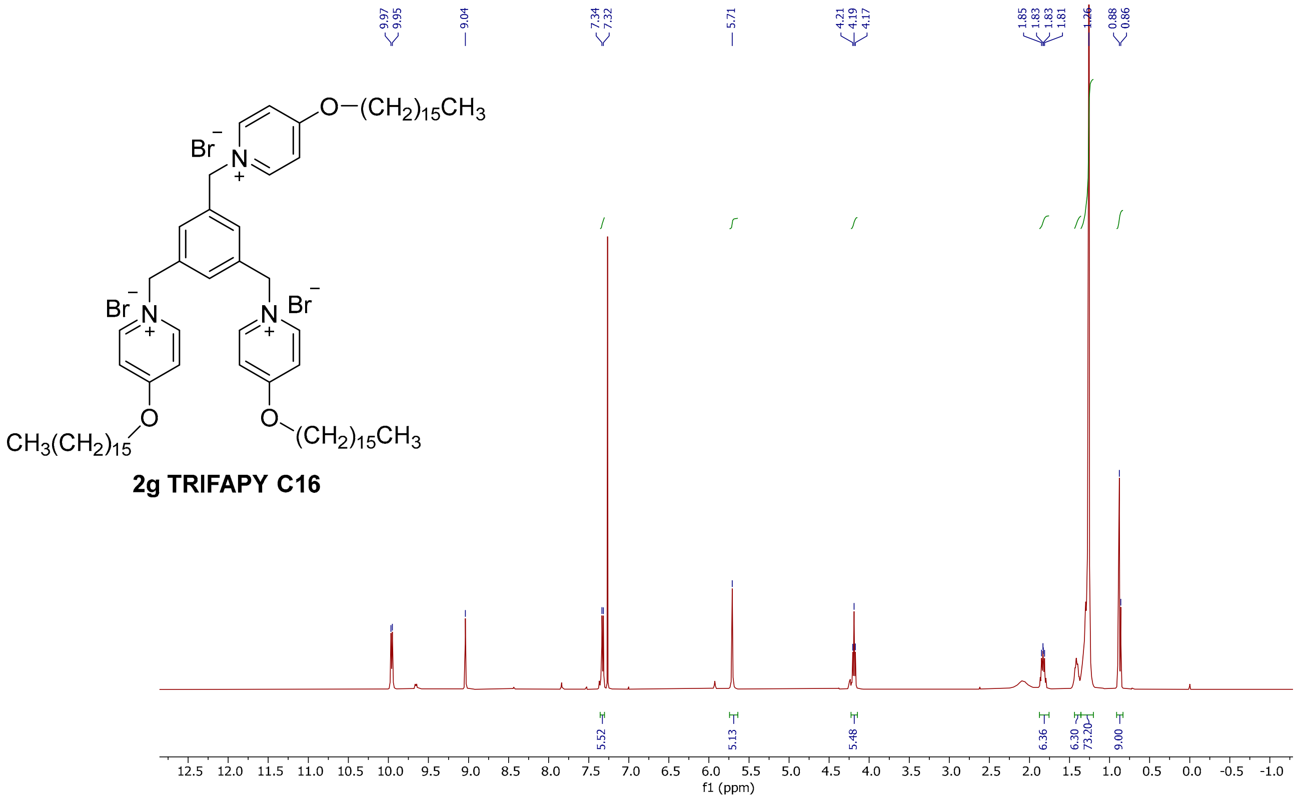

B.

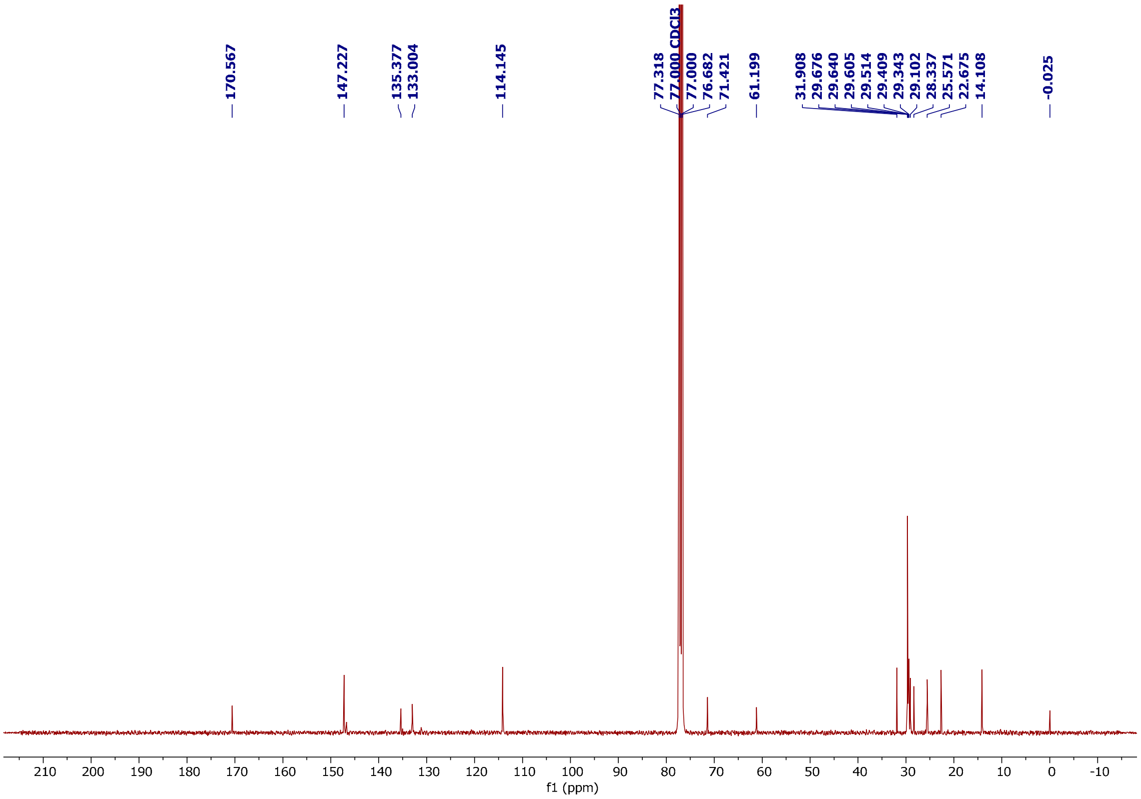

Figure S16. (A) ^{1}H-NMR (400 MHz, $CDCl_3$) and (B) ^{13}C NMR (100.6 MHz, $CDCl_3$) spectra of 1,3,5-Tris(4-hexadecyloxypyridiniomethyl)benzene tribromide.

A.

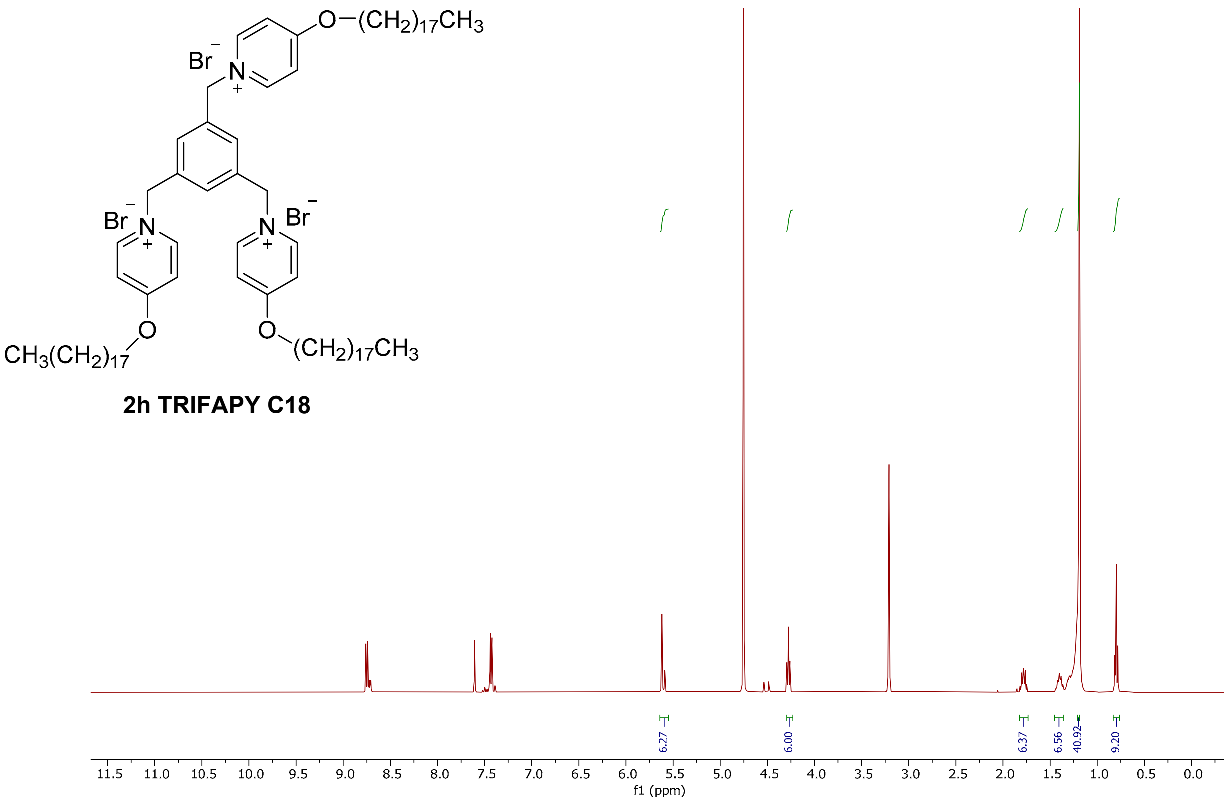

B.

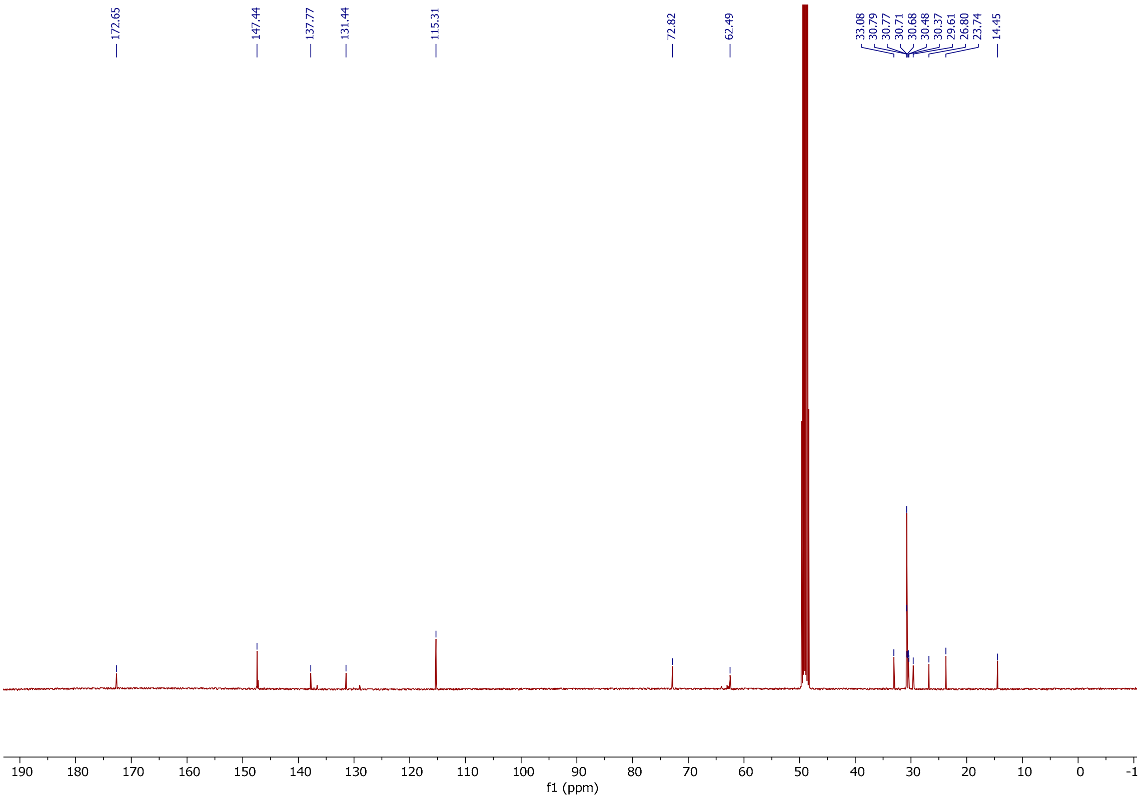

Figure S17. (A) ^{1}H-NMR (400 MHz, CD_3OD) and (B) ^{13}C NMR (100.6 MHz, CD_3OD) spectra of 1,3,5-Tris(4-octadecyloxypyridiniomethyl)benzene tribromide.

A.

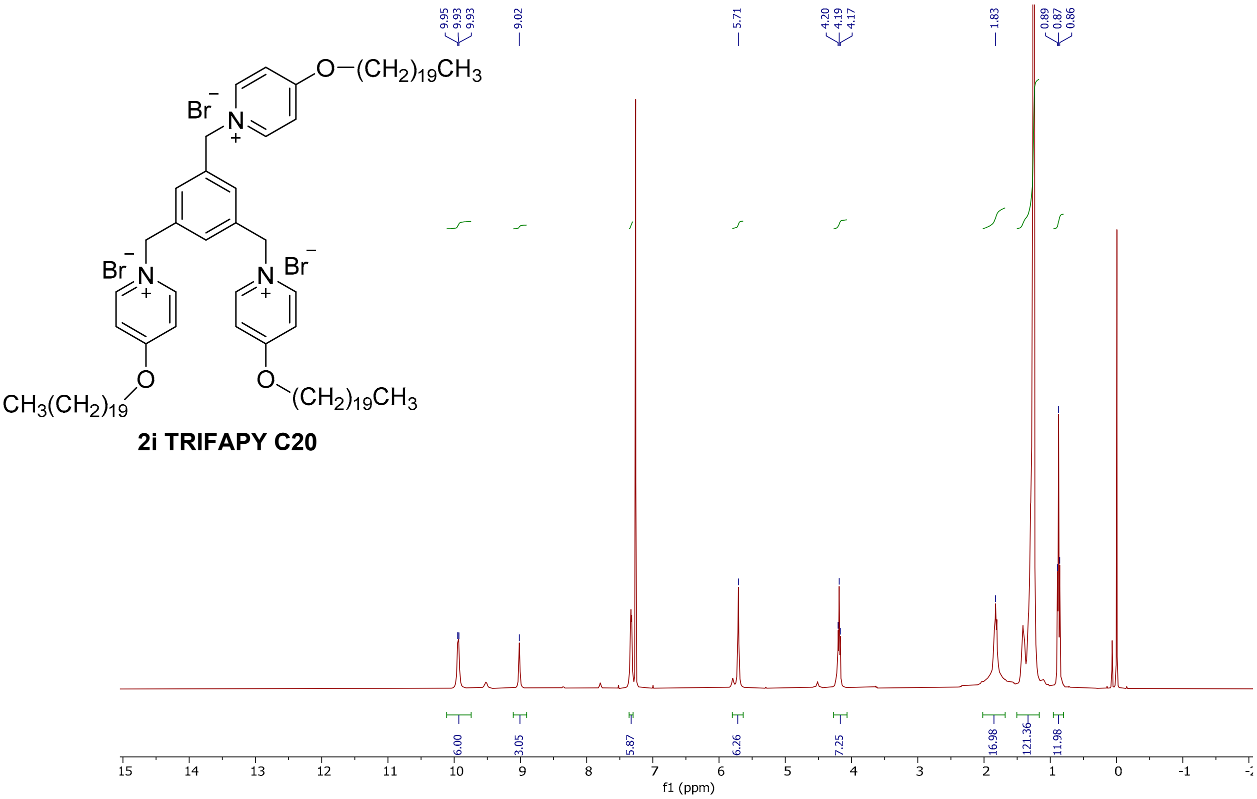

B.

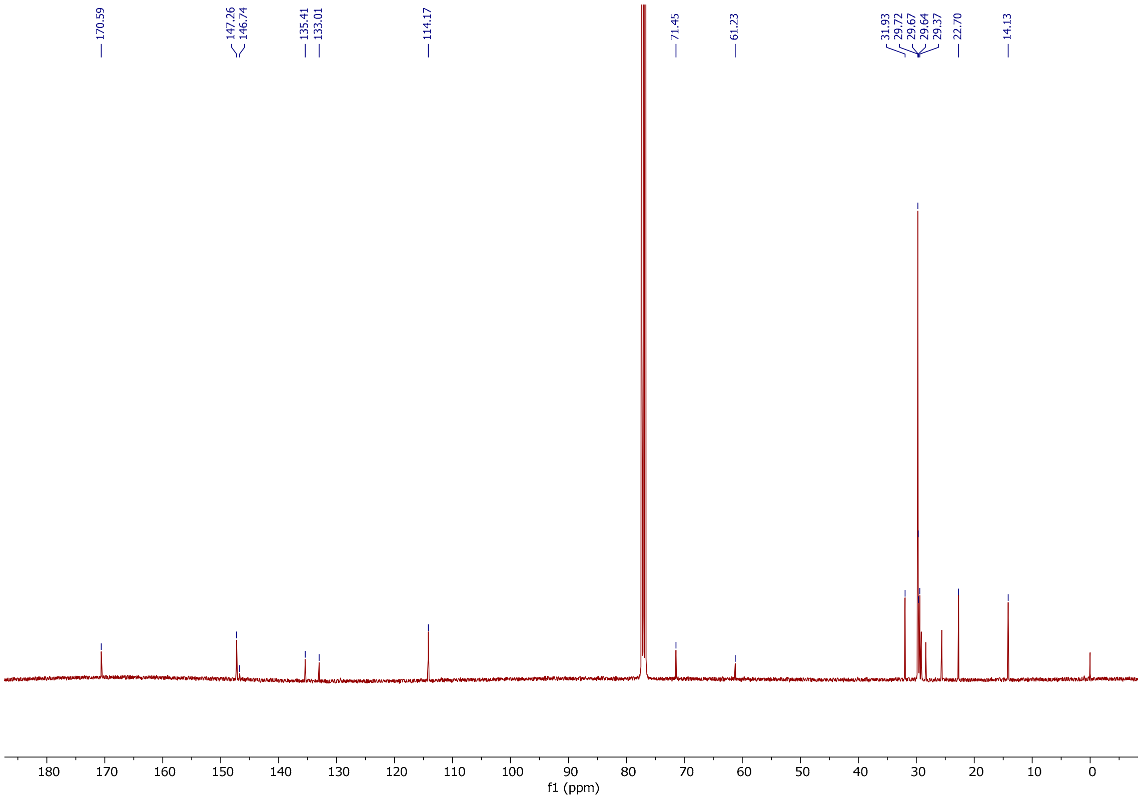

Figure S18. (A) ^{1}H-NMR (400 MHz, $CDCl_3$) and (B) ^{13}C NMR (100.6 MHz, $CDCl_3$) spectra of 1,3,5-Tris(4-eicosanyloxypyridiniomethyl)benzene tribromide.

A.

$CH_3(CH_2)_{18}CH_2OH$

3

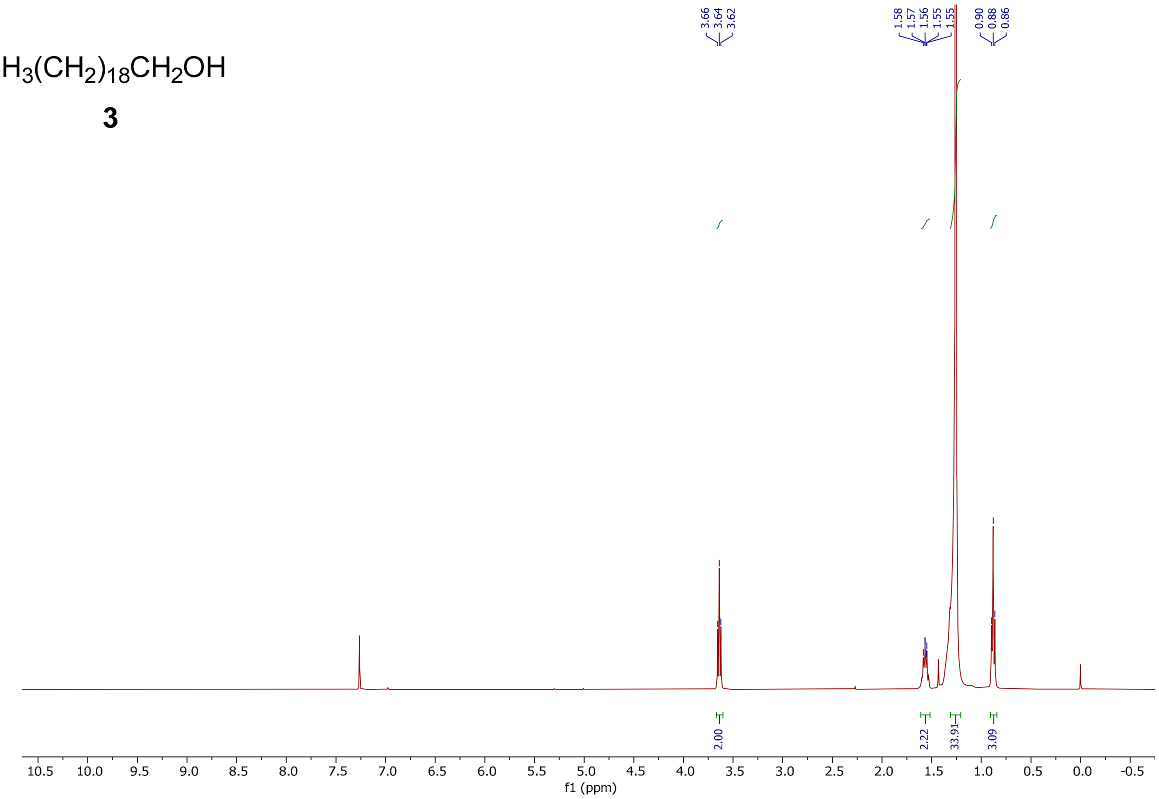

B.

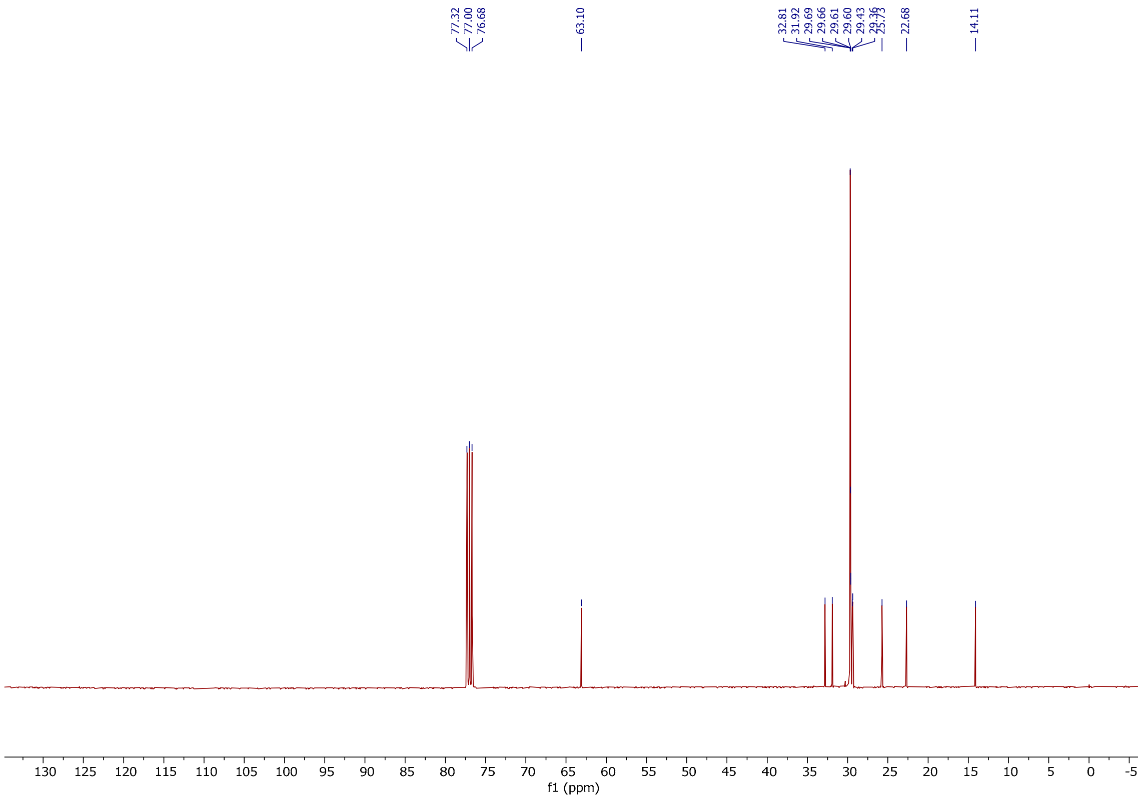

Figure S19. (A) ^{1}H-NMR (400 MHz, $CDCl_3$) and (B) ^{13}C NMR (100.6 MHz, $CDCl_3$) spectra of 1-Eicosanol.
